# An optimized approach for container deployment driven by a two-stage load balancing mechanism

**DOI:** 10.1371/journal.pone.0317039

**Published:** 2025-01-10

**Authors:** Chaoze Lu, Jianchao Zhou, Qifeng Zou

**Affiliations:** 1 School of Cyber Science and Engineering, Ningbo University of Technology, Ningbo, Zhejiang, China; 2 School of Humanities, Ningbo University of Finance and Economics, Ningbo, Zhejiang, China; University of Pisa, ITALY

## Abstract

Lightweight container technology has emerged as a fundamental component of cloud-native computing, with the deployment of containers and the balancing of loads on virtual machines representing significant challenges. This paper presents an optimization strategy for container deployment that consists of two stages: coarse-grained and fine-grained load balancing. In the initial stage, a greedy algorithm is employed for coarse-grained deployment, facilitating the distribution of container services across virtual machines in a balanced manner based on resource requests. The subsequent stage utilizes a genetic algorithm for fine-grained resource allocation, ensuring an equitable distribution of resources to each container service on a single virtual machine. This two-stage optimization enhances load balancing and resource utilization throughout the system. Empirical results indicate that this approach is more efficient and adaptable in comparison to the Grey Wolf Optimization (GWO) Algorithm, the Simulated Annealing (SA) Algorithm, and the GWO-SA Algorithm, significantly improving both resource utilization and load balancing performance on virtual machines.

## Introduction

Containerization technology [1, 2] is a lightweight form of operating system-level virtualization that packages and encapsulates applications along with their runtime dependencies into standardized, highly portable images. By isolating processes and enforcing resource constraints through container engines, this technology decouples applications from the underlying operating system and hardware, thereby allowing them to be packaged once and executed consistently across diverse environments. This technology presents several significant advantages, including decreased overhead, expedited startup times, enhanced portability, and strong support for contemporary development paradigms such as microservices and DevOps practices. These characteristics have established containerization as a fundamental technology for cloud-native applications and distributed architectures. Furthermore, containerization automates numerous facets of application management, including cluster monitoring and process-level data isolation, thereby improving scalability and performance. Cloud computing platforms have extensively embraced container technology to construct container-based clouds [3, 4], offering a comprehensive array of functionalities that streamline the development and operational processes. These platforms offer a range of functionalities, including container lifecycle management, resource scheduling, service orchestration, automated application deployment, monitoring and logging, configuration management, storage and network management, complemented by integrated security features. The adoption of container-based cloud solutions has markedly enhanced resource utilization, decreased operational costs, and accelerated business iteration cycles. By optimizing performance and providing a flexible, scalable environment, containerization is instrumental in improving user experience and facilitating enterprises in their digital transformation initiatives. This comprehensive ecosystem empowers organizations to efficiently develop, deploy, and manage containerized applications, thereby fostering innovation and operational agility.

In addition to being deployed in public clouds, private clouds, and various container service platforms, containers can also be deployed on physical or virtual hosts. This is the simplest and most prevalent method, particularly suitable for personal development, testing, and small-scale applications. Deploying containers on a single host entails the installation of widely-used container engine software, such as Docker or Podman, on either a physical or virtual machine, followed by the execution of containers within the container engine. This approach capitalizes on the isolation and security afforded by virtualization while also benefiting from the lightweight nature and flexibility inherent to containers. However, one common challenge associated with the containerized deployment model is the issue of uneven resource allocation. This phenomenon pertains to the disproportionate distribution of resources across various containers or virtual machines, which can result in suboptimal performance outcomes. In instances of uneven resource allocation, certain virtual machines may experience excessively high resource consumption, while others remain underutilized. Such imbalances can adversely affect the overall performance and stability of applications, particularly in contexts where precise resource allocation is critical [5]. The root causes of this issue can be attributed to inadequate virtual machine scheduling strategies, imbalanced workloads among containers, and resource performance of containerized workloads. These factors contribute to varied resource utilization across containers, ultimately impacting overall performance and resource utilization [6, 7].

In order to address the aforementioned issue, it is essential to investigate and develop more equitable and efficient resource allocation strategies in containerized deployments. Our main contributions of this study are as follows:

(1)The comprehensive deployment process for container services is categorized into two stages: the coarse-grained stage and the fine-grained stage. During the coarse-grained stage, the deployment of multiple container services across various virtual machines is optimized according to their resource requirements. During the fine-grained stage, resources on each virtual machine are distributed evenly among the container services.
(2)In the initial phase of addressing the challenge of distributing multiple containers across various virtual machines, we employ a greedy algorithm. Subsequently, to tackle the issue of resource allocation for multiple containers on a single virtual machine, we utilize a genetic algorithm. This approach enables us to derive a set of optimal resource allocation strategies that facilitate a reasonable and balanced distribution of resources among the containers on the virtual machines.
(3)Our approach addresses the limitations of traditional solutions, which primarily concentrate on the latter stages of the resource allocation problem while overlooking the overall balance in the initial stages. Furthermore, our approach underscores the significance of load balancing in the optimization of container service deployment.

This paper is organized as follows. “Background and related work” introduces related work. “Container deployment combinatorial optimization problem based on load balancing” introduces the two-stage partitioning situation and the mathematical modeling design of each stage. “Algorithm design for solving the two-stage container load balancing deployment model” introduces the research method and the specific algorithm design process of this paper. “Simulation experiment of container deployment optimization for two-stage load balancing” verifies and compares the deployment optimization method of this paper. “Summary and future work” summarizes the whole paper and introduces the future work.

## Background and related work

The challenge of rational container placement, deployment, and balanced resource allocation has attracted considerable attention from both industry and academia. Despite this interest, research in this domain remains predominantly exploratory and developmental. Resource allocation, in particular, constitutes a critical component of container deployment strategies. Researchers are concentrating on the development of intelligent algorithms that effectively manage resources such as CPU, memory, and network bandwidth. These algorithms must be aligned with application requirements to enhance overall system performance. By improving the performance, reliability, and resource utilization of container clusters, these advancements seek to optimize the efficiency and effectiveness of containerized environments.

Currently, both domestic and international researchers have extensively explored task balancing and placement issues, yielding notable results. For instance, Hirofuchi et al. [8] addressed the placement problem under various resource constraints, such as CPU and memory, by considering both the configuration time and migration time of virtual machines to optimize cloud resource allocation. Aslam et al. [9] provided a comprehensive review of load balancing algorithms in cloud environments from 2004 to 2015, categorizing these algorithms into two main types: static and dynamic. They provided detailed descriptions and comparisons of each algorithm, and evaluated the performance of different load balancing algorithms through multiple parameters such as fairness, response time, and throughput. Nakada et al. [10] utilized a genetic algorithm to develop effective placement strategies by adjusting parameter weights, though determining the optimal parameters for multi-objective optimization remains challenging. Patel et al. [11] proposed a dynamic priority spillover technique and introduced the concept of short-life and long-life containers to optimize resource utilization and address fragmentation issues in virtual machine (VM) placement. Xu et al. [12] approached the placement problem as a multi-objective optimization challenge, aiming to minimize total resource waste, power consumption, and cooling costs. They proposed an enhanced multi-objective optimization genetic algorithm to explore the solution space and reconcile conflicting objectives. Van et al. [13] defined the placement problem as a constraint satisfaction issue and employed constraint planning methods to model it, achieving an automated mechanism for virtual resource management. Dasgupta et al. [14] introduced a novel load balancing strategy based on a genetic algorithm (GA). This algorithm efficiently balances the load across cloud infrastructure while minimizing the makespan for a given set of tasks, demonstrating its effectiveness in optimizing resource allocation. It is noteworthy that machine learning technologies have demonstrated significant potential in addressing load balancing challenges [15]. However, for small-scale development users, the adoption of such technologies could significantly escalate development costs. Consequently, after comprehensive consideration, this paper has opted to employ heuristic optimization algorithms as the solution.

Resource allocation, a critical issue in cloud computing, involves distributing resources based on specific usage rules within a fixed resource environment and addressing fixed user resource requests. Numerous studies have proposed algorithms and solutions to optimize this process. Wei et al. [16] achieved preliminary independent optimization results using binary integer programming, reaching an ideal and fair resource allocation through evolutionary mechanisms. Their findings demonstrated the existence of Nash equilibrium in resource allocation games when feasible solutions are available. Lee et al. [17] developed a mechanism for adaptive and stable deployment on virtual machines, utilizing evolutionary game theory to model key performance parameters. However, their study focused solely on CPU performance and did not consider other critical parameters such as memory and I/O. Pandey et al. [18] approached resource allocation by modeling it as a directed acyclic graph and employed a particle swarm-based heuristic cloud resource scheduling algorithm. Their approach aimed to minimize computation and transmission costs effectively. Semmoud et al. [19] proposed a load balancing algorithm based on a starvation threshold, which ensures load balancing by activating at least one idle virtual machine. This method helps reduce overall migration costs and mitigates additional overhead. Shi et al. [20] introduced the BMin algorithm, an enhancement of the Min-min algorithm, and demonstrated its effectiveness through experiments using the CloudSim simulation framework. Their results indicated that the BMin algorithm improves throughput and resource load balancing, outperforming traditional algorithms. Shahid et al. [21] evaluated the performance of several existing load balancing algorithms, including Particle Swarm Optimization (PSO) and Round Robin (RR). They also explored various modeling and simulation methods for mobile cloud computing environments, which are essential for assessing cost and reliability trade-offs in a Pay-As-You-Go (PAYG) context [22].

In summary, the process of container deployment allows for the consideration of various perspectives and the application of diverse data analysis methods to allocate resources among containers on a single host. However, existing methodologies exhibit certain limitations and fail to achieve a balanced deployment from a comprehensive architectural viewpoint, as they lack unified optimization criteria. This paper introduces a two-stage optimization method for load balancing in container deployment, which aims to minimize the processing time of all subtasks as the primary optimization criterion. The first stage employs a greedy algorithm to facilitate the balanced deployment of virtual machines by effectively placing multiple container tasks. The second stage utilizes a genetic algorithm to achieve load balancing of container tasks through the allocation of resources on virtual machines. This approach is designed to enhance system responsiveness and improve resource utilization.

## Container deployment combinatorial optimization problem based on load balancing

### Fundamentals of container deployment

#### Microservice architecture

The concept of microservices was initially introduced by Martin Fowler and James Lewis in 2014. This architectural style entails decomposing an application into multiple small and independent service units, each capable of operating within its own process [23], and communicating via lightweight mechanisms such as HTTP APIs. Each service unit is developed autonomously and can be implemented using various programming languages, frameworks, and data storage technologies, provided that they conform to a standardized interface specification. These service units can be developed, tested, deployed, and scaled independently, without impacting the functionality of other service units, thereby collectively forming a complete application. The microservices architecture [24] offers developers an enhanced development paradigm for applications, characterized by:

(1) the independence of each microservice from one another;
(2) each microservice functioning as an atomic service that cannot be further subdivided;
(3) the capacity of microservices to be rapidly combined and refactored into a cohesive system.

#### Virtual machine technology

A virtual machine is a software technology that simulates hardware and operating systems. It abstracts, transforms, and partitions the hardware resources of a computer, providing multiple virtual execution environments. As a result, it enables running multiple different operating systems on a single physical machine. The concept of virtual machines was first introduced by IBM in the 1960s implement multi-user and multitasking capabilities for large-scale computers. Later, with the development of personal computers and the internet, virtual machine technology has been widely applied and developed. Its fundamental principle involves creating one or more virtual hardware environments, known as virtual machines [25], on the hardware resources of a physical machine through a software layer called a virtualization layer or hypervisor. The virtualization layer manages and allocates the CPU, memory, disk, network, and other resources of the physical machine to each virtual machine. It also provides a set of standard interfaces and protocols for communication and interaction between virtual machines and the physical machine.

Virtual machine technology provides infrastructure-level support and advantages for microservices architecture, helping to achieve isolation, elastic scalability, management deployment, and a more flexible and diverse selection of runtime environments.

#### Containerization technology and deployment

The concept of containers can be traced back to the Unix system call mechanism known as chroot (Change Root), which was proposed in 1979. Chroot facilitates the creation of an isolated environment that restricts access permissions for specific users or services, thereby preventing processes from accessing or modifying files and resources outside the designated directory tree. This methodology for process restriction and isolation served as a foundational element for the evolution of container technology [26]. Subsequently, the Linux kernel introduced namespaces and control groups (cgroups) to enhance process isolation and resource management. In 2008, the Linux Container Project launched the Linux container engine. This was followed by the introduction of the Docker container engine in 2013, which addressed issues related to standardization and portability within the realm of containerization. The advent and advancement of technologies such as the Kubernetes container orchestration platform in 2014, which is capable of managing distributed container clusters, signified the official emergence of containerization technology into a phase characterized by rapid technological growth.

Container technology represents a lightweight virtualization approach that consolidates all necessary files, libraries, dependencies, and other requirements essential for executing a software program. This technology can be deployed across various computing environments, including physical servers, virtual machines, and cloud servers. Container technology facilitates environment isolation and resource limitation, providing process-level isolation by creating an independent runtime environment for a collection of processes. Moreover, it enables applications to be deployed across diverse runtime environments, allowing them to operate independently in separate spaces without compromising the performance of other applications, even in the presence of errors. This characteristic enhances portability and fault tolerance for software developers. Additionally, containerization supports rapid updates and releases of software programs through the use of container modules, without disrupting or affecting the operating system or other applications. Consequently, it fosters agility and scalability while ensuring compatibility. Thus, container technology complements microservices architecture by promoting decoupling, high scalability, and flexibility in the implementation of microservices.

**Fig 1:**
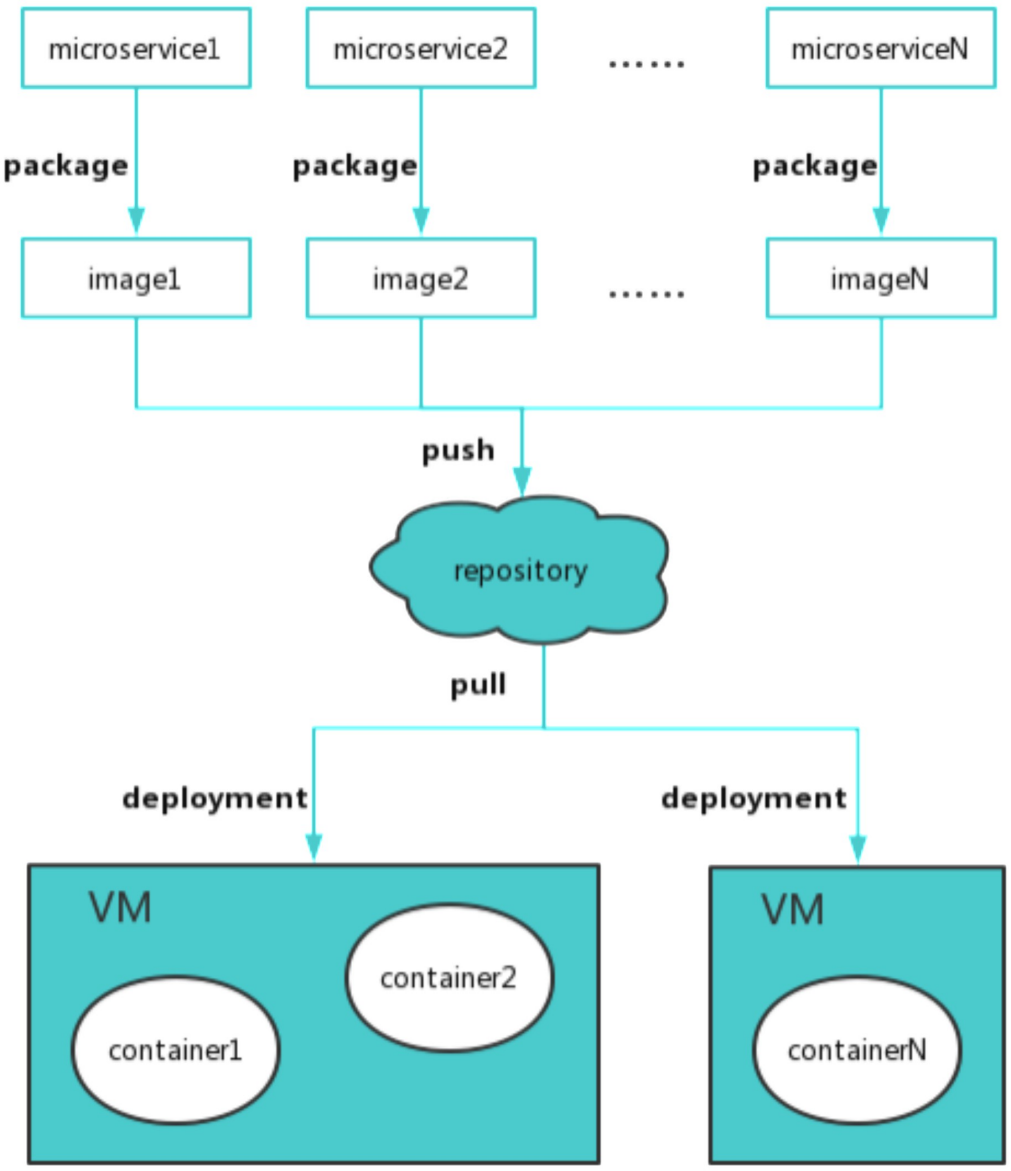
Containerization deployment flowchart.

Due to the ability to run each microservice in an independent container, developers package the microservices and their dependencies into a container image, which can then be deployed on a single virtual machine. The general solution design consists of four main steps: application packaging, image building, image pushing, and container orchestration management [27], as shown in Fig 1. Application packaging can be configured within the configuration file located in the root directory of the project to facilitate the automatic packaging of the application. The process of image building can be accomplished by creating a Dockerfile and utilizing the build command. The Dockerfile encompasses various instructions and specifications essential for constructing the image, rendering it more efficient and concise in comparison to manual image building methods. Once the image has been constructed, it is subsequently pushed to a repository for storage, enabling it to be retrieved at any time for the establishment of a development environment. As the number of containers increases, it becomes imperative to manage and configure the operational order, communication, and status among the containers. Consequently, a container orchestration tool is required for the effective management and scheduling of containers. Prominent orchestration tools available in the market include Kubernetes, Docker Compose, and Docker Swarm.

### Two-stage load balancing container deployment problem model

Based on the potential issues that may arise from the aforementioned deployment process, this paper researches the problem of two-stage load balancing container deployment optimization with coarse-to-fine granularity. In order to describe the problem more clearly and in detail, this paper divides this optimization problem into two parts. The first part is to place the container requests, which require certain resources, onto multiple virtual machines in a reasonable manner. The second part is to further allocate the inherent resources on the virtual machines to the respective container requests in a balanced way. The problem is further described as follows.

#### Container placement problem among virtual machines

**Fig 2:**
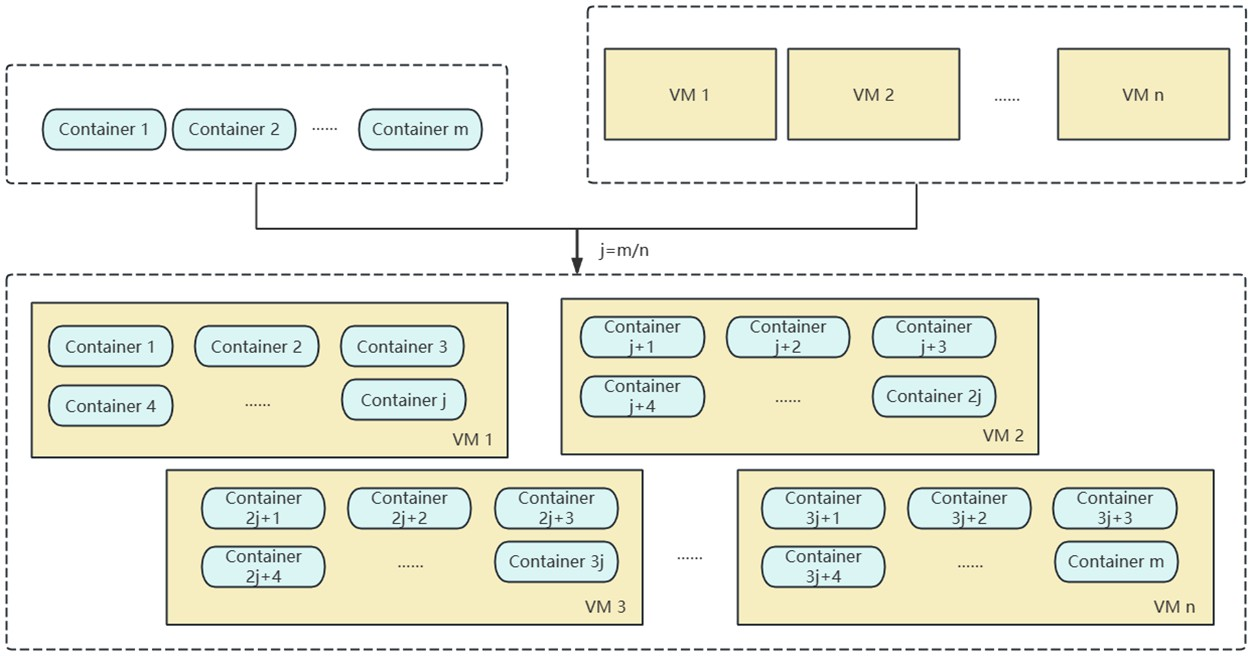
The possible states of virtual machines deployed using the principle of average allocation.

The objective of this study is to deploy a specified number of containers onto a cluster of virtual machines (VMs). This process, which involves the allocation and deployment of multiple containers across the VM cluster, is commonly referred to as the container placement problem. In the allocation and deployment strategy, if an even distribution approach is adopted, where containers are deployed evenly across VMs based on their quantity. However, it is important to note that each container possesses distinct resource requirements; some containers may necessitate a substantial portion of a VM’s resources, while others may require significantly less. Consequently, an even distribution based solely on quantity may result in certain VMs being disproportionately burdened with tasks, while others remain relatively underutilized. This phenomenon leads to a concentration of containers, as illustrated in Fig 2.

Therefore, in order to solve this problem, this paper should deploy containers reasonably, so that the available virtual machines can handle the work, and make full use of the resources of multiple machines to make the containers run more quickly and smoothly.

**Definition 1(Coarse-grained Way):** Suppose there are *n* virtual machines, each with distinct configurations, and *m* different container deployment requests, alongside *m* container task requests that must be allocated to *n* virtual machines. It is stipulated that the total amount of resources required by each container task request is denoted as *CT*_*SUM*_*i*_(1≤i≤m), The deployment of containers to the virtual machines occurs in a sequential manner. The objective is to deploy a certain number of containers evenly across the virtual machines. Each virtual machine should have a resource consumption within the range [*Res*_*min*_, *Res*_*max*_]. This approach guarantees that no virtual machine is either overloaded or left idle.

The parameters in the research problem model are now defined and explained, and the objective function and related constraints are shown in Table 1.

**Table 1:** Parameter meaning for coarse-grained stages.

Parameter	Description
CT{CT_1_,CT_2_,…,CT_*m*_}	Container request deployment set
VM{VM_1_,VM_2_,…,VM_*n*_}	Available virtual machine set
*CT*_*SUM*_*i*_(1≤i≤m)	The total amount of resources requested by *CT*_*i*_
*VM*_*SUM*_*j*_(1≤j≤n)	The total amount of resources consumed by *VM*_*j*_
*Employ*_*i*,*j*_	0-1 variable, whether *CT*_*i*_ is deployed to *VM*_*j*_, if yes, then 1, if not, then 0

This problem aims to place each container on a suitable virtual machine without causing congestion. The objective function values are the endpoints of the range [*Res*_*min*_, *Res*_*max*_], which represent the resource consumption of each virtual machine after allocation. The method of determining the values of *Res*_*min*_ and *Res*_*max*_ is as follows:

$$\begin{array}{c} Res_{min}=\frac{1}{n}\sum_{i=1}^{m}CT\_SUM_{i}-\left(Max\left\{CT\_SUM_{1,2,...,m}\right\}+Min\left\{CT\_SUM_{1,2,...,m}\right\}\right)/n \end{array}$$ (1)

$$\begin{array}{c} Res_{max}=\frac{1}{n}\sum_{i=1}^{m}CT\_SUM_{i}+\left(Max\left\{CT\_SUM_{1,2,...,m}\right\}+Min\left\{CT\_SUM_{1,2,...,m}\right\}\right)/n \end{array}$$ (2)

The constraints are as follows:

$$\begin{array}{c} Res_{min}\leq VM\_SUM_{j}\leq Res_{max},1\leq j\leq n \end{array}$$ (3)

$$\begin{array}{c} \sum_{i=1}^{m}\sum_{j=1}^{n}Employ_{i,j}=1 \end{array}$$ (4)

*Res*_*min*_ and *Res*_*max*_ are the minimum and maximum values of the reasonable resource range for each virtual machine *VM*_*j*_(1≤j≤n). They depend on the current number of container requests that need to be deployed. They depend on the number of current container requests that need to be deployed, and then reflect load balancing by taking into account the maximum and minimum resource requirements for deploying containers and measuring the extreme differences in the rational allocation of resources by VMs. Eq 4 indicates that a container request can only be deployed to one virtual machine.

#### Internal resource allocation problem of virtual machines

From the above problems, it can be concluded that m different container requests have been reasonably allocated to n virtual machines. However, this allocation does not guarantee that the container requests deployed on each virtual machine will be able to complete their tasks efficiently and concurrently. Given that each container is associated with varying task workloads, some containers necessitate substantial amounts of CPU, memory, and computational time, while others require only minimal CPU and memory resources to achieve rapid completion. The overall completion of a subprocess is contingent upon the successful execution of all container threads within that subprocess. Even if certain container threads have concluded their tasks, they must still await the completion of other threads that remain unfinished. These subprocesses typically arise from complex and large-scale services. Consequently, it is imperative to develop an optimization function that effectively balances the allocation of diverse resources, thereby facilitating the concurrent operation of containers on virtual machines while minimizing overall time consumption.

**Definition 2(Fine-grained Way):** Suppose *w* containers are deployed on a virtual machine, and each container request *i* has a different resource type request. It also states that the entire task is considered complete only when all containers on the virtual machine have completed their work.

**Fig 3:**
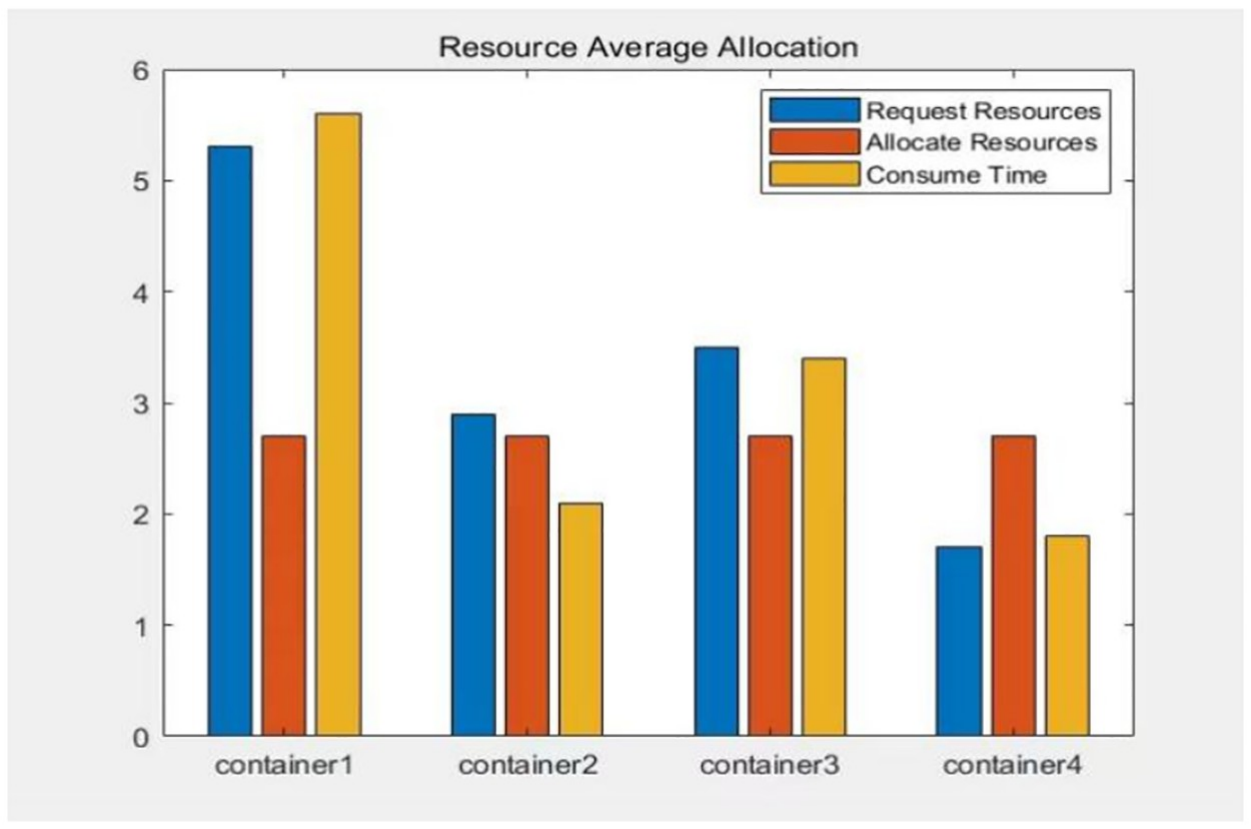
The average allocation time of virtual machine resources.

**Fig 4:**
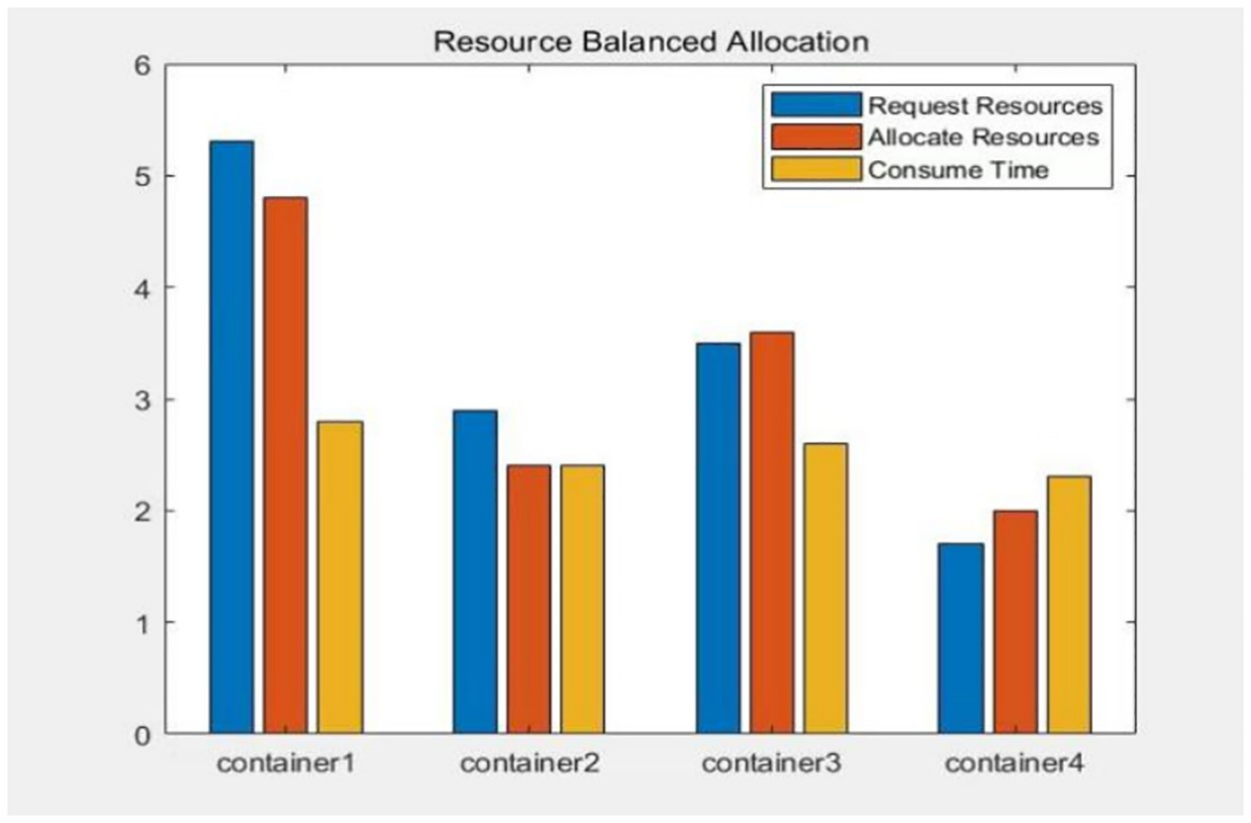
The balanced allocation time of virtual machine resources.

In order to meet this condition: assuming that when each container request is allocated a different amount of resources, the completion time of the process also varies. Container *a* will have a shorter completion time if it receives sufficient resources, while container *b* will have a longer completion time if it is allocated insufficient resources. However, even after container a finishes its process, it still needs to wait for container b to complete its task. Therefore, if we distribute all resources evenly among the containers based on the number of container requests, the above-mentioned unreasonable situation will occur, as shown in Fig 3. A reasonable approach is to balance the resource allocation of the virtual machines. This way, each container thread can work with a fair share of resources and finish at similar times. This will minimize the overall completion time of the subprocess task, as shown in Fig 4.

The parameters in the research problem model will now be defined and explained. The objective function and related constraints are shown in Table 2.

**Table 2:** Parameter meaning for fine-grained stages.

Parameter	Description
*CT*_*ASSET*_*i*_{*c*_*i*1_, *c*_*i*2_, …, *c*_*ir*_}	The resource set required by container i during process execution
*VM*_*ASSET*_*i*_{*v*_*i*1_, *v*_*i*2_, …, *v*_*ir*_}	The resource set provided to container i by the virtual machine
*T*_*MIN*_*i*_	The minimum time required for container i to complete a process
*T*_*MAX*_*i*_	The maximum time required for container i to complete a process

In this problem, it is assumed that only three types of resources are studied: CPU, memory, and I/O, Hence *r* is equal to 3. To minimize the completion time of the entire task on the virtual machine, resources should be evenly distributed and all container processes should work in parallel. The formula required for the objective function is as follows:

$$\begin{array}{c} U_{i}=\frac{1}{r}\sum_{k=1}^{r}Min\left(1-\frac{c_{k}-v_{k}}{c_{k}},1\right) \end{array}$$ (5)

$$\begin{array}{c} T_{i}=T\_MIN_{i}-\left(T\_MIN_{i}-T\_MAX_{i}\right)*U_{i} \end{array}$$ (6)

The formulated objective function is as follows:

$$\begin{array}{c} G\left(T\right)=\frac{1}{w}\sum_{i=1}^{w}{\left(T_{i}-\frac{1}{w}\sum_{i=1}^{w}T_{j}\right)}^{2} \end{array}$$ (7)

The formulated constraints are as follows:

$$\begin{array}{c} \forall x\in v_{i},x>0 \end{array}$$ (8)

*U*_*i*_ is the utilization rate of container request *i*. It measures how well the virtual machine allocates three types of resources to container request *i*. It is calculated by standardizing the difference between the container request *i* and the resource allocation. The virtual machine may provide more of a certain resource than container request *i* needs. In that case, container request *i* can use that resource fully in an ideal state. The utilization rate of that resource is 1. *T*_*i*_ represents the time required for container *i* to complete its processes after being allocated different amounts of various resources by the virtual machine. The utilization rate *U*_*i*_ of container *i* can be used to determine the amount of resources allocated based on the size of the workload. The more the allocated resources satisfy the resource requirements of container *i*, the more efficiently it can complete its tasks, and the closer the completion time is to the minimum time required by the thread. Conversely, if the allocated resources do not meet the resource requirements of container *i*, the opposite is true. The objective function *G*(*T*) measures the dispersion of the completion times of each container thread. The resource allocation is more effective when the times are similar and the data deviation is low. This means that faster threads do not have to wait for slower threads to finish. Eq 8 indicates that each container request must receive resources from the virtual machine, and there should be no situation of resource allocation failure.

## Algorithm design for solving the two-stage container load balancing deployment model

This paper divides container deployment into two-stage problems. The first problem is how to place container service requests on virtual machines in a reasonable manner. The second problem is how to allocate resources to virtual machines with deployed container services, so that each container service can complete tasks relatively quickly and improve overall service efficiency. The following are the method strategies proposed in this paper to solve these two problems.

### Greedy strategy for solving the container placement problem among virtual machines

The first problem to be addressed involves the strategic deployment of each container to the appropriate virtual machine. Given that different container tasks exhibit varying resource consumption levels on virtual machines, it is impractical to pre-allocate containers to specific virtual machines. Consequently, it is essential to first sort the container tasks in descending order based on the time they require. The advantage of this sorting is that it can use the greedy algorithm [28], which enables the identification of a locally optimal solution at each selection step, thereby allowing for the rapid approximation of an optimal solution. To achieve an effective deployment strategy, container tasks that demand the most time should be assigned to the virtual machine currently exhibiting the lowest resource usage. The pseudocode of the algorithm is shown in Algorithm 1. This approach promotes a balanced distribution of resource consumption across all virtual machines executing the container services. The operational process is illustrated below.

Step 1: For the container structure list, sort the container list in descending order according to the amount of requested resources;
Step 2: Remove the first container from the container list. The container that requests the most resources is allocated to the first VM in the VM list, that is, the VM that consumes the least resources.
Step 3: Deploy the corresponding container task to the corresponding VM, and sort the VM list in ascending order based on the resource consumption.
Step 4: Repeat Steps 2-3 until all containers in the container list are assigned to the virtual machine.

**Algorithm 1 Greedy Algorithm for the Container Placement**

**Require**: List of containers CT[ ], list of virtual machines VM[ ].

**Ensure**: An allocation deployment plan.

1: Initialize CT=[ ]

2: Initialize VM=[ ]

3: Sort CT[ ] in descending order by time

4: **while** tasks is not empty **do**

5:  Sort VM[ ] in ascending order by resource consumption

6:  Assign the last container in CT[ ] to the first vm in VM[ ]

7:  Remove the last container from CT[ ]

8: **end while**

9: **while** true **do**

10:  Find the vm with the least resource consumption (minVm)

11:  Find the vm with the most resource consumption (maxVm)

12:  **if** the difference between maxVm and minVm resource consumption <= Q_max − Q_min **then**

13:   break the loop

14:  **else**

15:   Remove the last container from maxVm’s task list

16:   Assign this container to minVm

17:   Update maxVm’s time and resource consumption

18:  **end if**

19: **end while**

20: **return** allocation deployment plan

### Genetic algorithm for solving virtual machine internal resource allocation problem

#### Generic load balancing techniques

Generic load balancing techniques [29], such as Round Robin, First-Come-First-Served(FCFS), Min-Min and Max-Min algorithms, were once widely used in cloud computing environments. However, with the increasing demand for resources and the diversity of demands, these traditional load balancing methods gradually show their limitations when facing complex resource allocation scenarios. For example, Round Robin simply assigns requests to each server in turn; FCFS, while simple and fair, does not take into account the execution time of the tasks, which may lead to longer average waiting and response times; the Min-Min algorithm prioritises scheduling of the smallest tasks, which results in larger tasks being delayed until the end, thus lengthening the completion time; and the Max-Min algorithm prioritises scheduling of the largest tasks but leads to load imbalance when tasks are executed at similar times. These methods cannot be dynamically adjusted to adapt to changing environments and task types, so more advanced load balancing techniques are needed to meet this challenge. For this reason, load balancing techniques based on natural phenomena have emerged, such as ant colony algorithms, bee algorithms and genetic algorithms. In this paper, we will focus on the genetic algorithm [30], an algorithm that performs well in dealing with extensive search spaces and complex objective functions. The main advantages of genetic algorithms include their ability to effectively avoid falling into local optimal solutions and their strong adaptability to cope with complex problems. Next, the fundamentals of genetic algorithms and their application in load balancing are described in detail.

#### The fundamental concept of genetic algorithm

Load balancing is a key issue in distributed systems, aiming to evenly distribute workloads across multiple computing resources to improve the overall performance and reliability of the system. Load balancing is particularly important in cloud computing environments because it directly affects resource utilization and quality of service. However, the load balancing problem is highly complex and dynamic because the resource demand and system state may change at any time. Traditional load balancing methods often struggle to cope with these challenges, especially when global optimization is required.

Genetic algorithms [31] are adaptive global optimization methods that mimic natural selection and genetic mechanisms. These algorithms are based on Darwinian evolution and Mendelian genetics and consider all individuals in a population as search entities. Genetic algorithms use stochastic optimization techniques to efficiently explore the parameter space and dynamically learn and update their understanding of the problem space during the search process. They are able to adaptively adjust the search strategy to find the optimal solution. Meanwhile genetic algorithms exhibit excellent global search capabilities in resource allocation and load balancing problems. They can optimize multiple load balancing metrics, which is highly compatible with the multi-objective nature of the load balancing problem, and are scalable, allowing the integration of new genetic operations or parameter tuning based on specific load balancing requirements, adapting to the dynamic nature of the load balancing problem. In addition, genetic algorithms are remarkably flexible and robust to the dynamics and uncertainties in load balancing problems. Therefore, genetic algorithms have significant advantages and potential in solving complex load balancing problems.

#### Algorithm design for the optimization model

*(1) Encoding method.* Common encoding methods used in genetic algorithms include binary encoding, gray code encoding, real number encoding, etc. Binary encoding is simple and straightforward to implement, and can swiftly accomplish encoding and decoding in genetic algorithm operations. However, it also has some limitations, such as requiring longer bit strings to enhance the accuracy of the solution, which leads to an expanded solution space and a decreased algorithm efficiency. At the same time, binary encoding also cannot reflect the relationship between variables. Therefore, this paper chooses the real number encoding method, which can improve the calculation accuracy, does not need to convert the base, and is suitable for numerical optimization problems. This paper takes the amount of resources allocated by each virtual machine to the container as the gene value.

**Fig 5:**
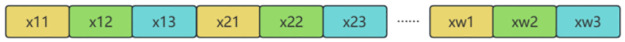
Chromosome composition designed by genetic algorithm.

**Definition 3(Chromosome):** Assuming that *w* container services are deployed on a virtual machine, each container service will request resources of *c*_1_,*c*_2_,…,*c*_*k*_ respectively, then the chromosome encoding is: *X* = *x*_11_,*x*_12_,…,*x*_1*k*_,*x*_21_, *x*_22_,…,*x*_2*k*_,…,*x*_*w*1_,*x*_*w*2_,…,*x*_*wk*_, since this paper assumes that only CPU, memory, and I/O are studied. These three types of resources, therefore, the composition of a chromosome is shown in Fig 5, where *x*_11_,*x*_12_,*x*_13_ represent the three types of resource requests required by the first container on a virtual machine.

*(2) Population setting*. Before performing the algorithm, the initial population and the population size must be set first. According to the actual situation, the initial population can be set in the possible distribution range of the entire problem domain. The method of initializing the population individuals in this paper is to randomly generate n genes within the specified resource range to form a chromosome, that is, a chromosome is allocated. The sum of *x*_11_,*x*_21_,…,*x*_*w*1_,*x*_12_,*x*_22_,…,*x*_*w*2_ and *x*_13_,*x*_23_,…,*x*_*w*3_ values will not exceed the corresponding total amount of resources on the virtual machine, and the initialization range of gene values is set according to different resource amounts. The population size is closely related to the execution efficiency and final result of the genetic algorithm. When the population size is small, the optimization performance obtained by the genetic algorithm will not be very good. When the population size is large, the computational complexity will also increase accordingly. This paper sets the population size NP to 600 and the number of iterations to 200. This scale dimension is moderate.

*(3) Fitness function*. Fitness refers to the ability of individuals in a biological population to adapt to the living environment. In genetic algorithms, the mathematical function used to evaluate the quality of individuals is called the fitness function of individuals. The fitness function has a direct impact on the performance of the genetic algorithm. Therefore, this paper defines the fitness function as the objective function of the original problem, and computes the resource utilization rate *U*_*i*_ and running time *T*_*i*_ of each individual in each generation of the population based on Eqs 5 and 6, and then proceeds to calculate using Eq 7. The individual with the smallest variance of running time in each generation of the population is taken as the optimal solution, that is

$$\begin{array}{c} G\left(T\right)=\frac{1}{w}\sum_{i=1}^{w}{\left(T_{i}-\frac{1}{w}\sum_{i=1}^{w}T_{j}\right)}^{2} \end{array}$$ (7)

*(4) Genetic operator design*. *Selection operator*: The function of the selection operator is to select good individuals from a certain generation of population and inherit them to the next generation of population. This paper adopts the roulette wheel selection algorithm [23]. The underlying principle of the roulette wheel selection algorithm is that individuals with higher fitness have a higher chance of being selected, but not necessarily. It also allows individuals with lower fitness to have some opportunity of selection, thus ensuring survival of the fittest. This paper calculates the fitness value of each individual for a certain generation of population with a size of NP, and adds up all the fitness values to obtain the total fitness value. The selection probability of an individual is calculated as individual fitness value/total fitness value. Then, a random number R between 0 and 1 is generated. Next, the selection probabilities of each individual are added up until the sum exceeds R. The individual corresponding to that sum is selected. In order to ensure the diversity of the population, repeat this step until NP individuals are selected to generate the next generation.

**Fig 6:**
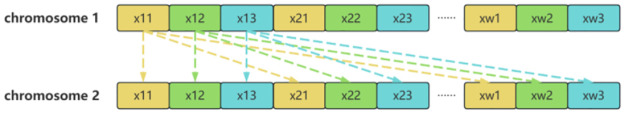
The same type of resources of two chromosomes can be crossed.

*Crossover operator*: Genetic algorithm employs crossover operator to alter the structure of two chromosomes, swapping gene segments between them, in order to modify the gene structure and enhance the search capability. This paper randomly selects two chromosomes, and randomly picks a position between the chromosome lengths to exchange with the corresponding exchangeable position of another chromosome (the same resource type in the chromosome is the corresponding exchangeable position), for example, *x*_11_ in chromosome 1 can be exchanged with any value of *x*_11_,*x*_21_,…,*x*_*w*1_ in chromosome 2, as shown in Fig 6.

*Mutation operator*: Genetic algorithm uses mutation operator to change the gene structure of a single individual, so that the new gene structure is closer to the optimal solution. For a certain individual in the population, some genes on a certain gene or some genes are changed to other alleles with a certain probability (mutation probability). This paper applies real number mutation, which means that after the crossover operation, the gene value of each individual in the population is substituted by a random value within the value range, taking into account the mutation probability. Only after exceeding a certain probability, mutation may occur. Otherwise, mutation will develop into a random search situation. After completing the crossover operation in this paper, a random number rand between 0 and 1 will be generated. If rand is less than the mutation rate *α* defined in this paper, which is 0.4, then the mutation operation will be executed, and *x*_11_,*x*_21_,…,*x*_*w*1_,*x*_12_,*x*_22_,…,*x*_*w*2_ and *x*_13_,*x*_23_,…,*x*_*w*3_ will undergo mutation within the given range, making sure that the total of the three kinds of resource requests does not surpass the overall amount of resources of the virtual machine.

*Proposition*: Let the decision vector *X* be utilized to construct the solution space for the problem within the context of the biological evolution process as implemented in genetic algorithms.

**Fig 7:**
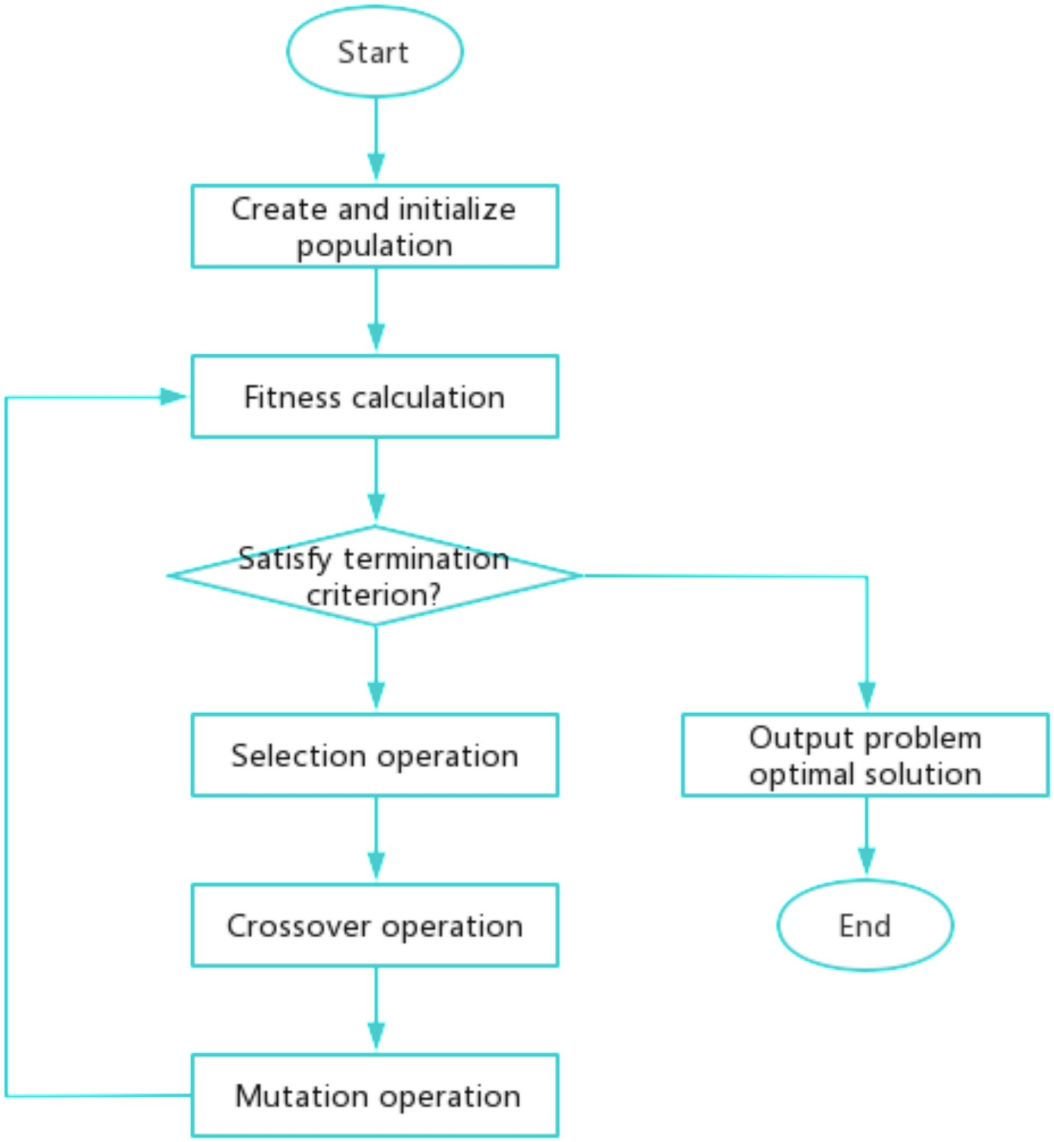
Genetic algorithm flow chart.

Proof: Through a series of genetic operations, including gene crossover and mutation among chromosomes, multiple iterations are conducted. The flowchart is shown in Fig 7. In accordance with the principle of survival of the fittest, individuals exhibiting higher fitness levels are selected for inheritance across generations. This process ultimately leads to the identification of a solution that either reaches or approximates the optimal solution to the problem at hand. The pseudocode of the algorithm is shown in Algorithm 2.

**Algorithm 2 Genetic algorithm for virtual machine internal resource allocation**

**Require**: Container resource requirements D[ ], Total virtual machine resources V[ ].

**Ensure**: An optimal set of resource allocation options. operations

1: Define genetic algorithm parameters(Population size NP, Maximum number of generations G, Chromosome length N)

2: Initialize population

3: Initialize generation counter

4: **while** generation counter < G **do**

5:  Calculate fitness for each individual in population

6:  Record best fitness for this generation

7:  Individuals in the population are selected using the roulette selection method

8:  crossover_idx1 = random integer between 1 and N

9:  crossover_idx2 = random integer between 1 and N

10:  Perform crossover based on idx1 and idx2

11:  mutation_idx = random integer between 1 and N

12:  Perform mutation based on mutation_idx

13:  Increment generation counter

14: **end while**

15: **return** an optimal set of resource allocation options

*(5) Time complexity analysis*. In this paper, we analyze the time complexity of using genetic algorithm to allocate resources for load balancing, which proves the rationality and application value of the algorithm. The main process of genetic algorithm includes calculating the fitness function, selection, crossover and mutation operations. In calculating the fitness function, the time complexity is O(NP·w), and the fitness values of w individuals in NP populations are calculated separately. In the selection operation, the time complexity is O(NP), and the population with the large fitness value is selected among the NP populations. In crossover operation, the time complexity is (NP·r), two individuals are randomly selected among the NP populations, and then two-by-two exchanges are performed on r resource types. In the mutation operation, the time complexity is O(NP·r), and any one of the r kinds of resources of an individual is randomly selected in the NP populations for mutation. Where NP is the number of population size individuals, w is the number of individuals within each population (i.e., the number of containers), and r is the number of resource types required for each container. Iterate the above operation until the maximum number of iterations G is satisfied before stopping, so the total time complexity is shown below.

$$\begin{array}{c} Time\;complexity=O(G*(NP*w)+NP+NP*r) \end{array}$$ (9)

## Simulation experiment of container deployment optimization for two-stage load balancing

Upon the completion of the modeling and design of the theoretical knowledge component, this paper conducts simulation experiments (https://github.com/buer33/Greedy-Genetic-Algorithm) focused on the optimal allocation of multiple container request tasks and the reduction of the time required for each subprocess task. The initial phase of the experiment validates that the greedy algorithm effectively assigns each container request task to an appropriate virtual machine based on the resource requirements of each task. The results indicate that this approach significantly outperforms the average resource consumption associated with randomly allocated virtual machines. Subsequently, the study demonstrates that the genetic algorithm can allocate the intrinsic resources of the virtual machine in accordance with the resource requirements and completion times of each container request task. This allocation strategy aims to minimize the completion time of each container thread, thereby expediting the completion of subprocess tasks, and the findings reveal that this method yields a more equitable distribution of resources compared to average allocation. Finally, the paper compares the running time differences between the greedy-genetic algorithm and other algorithms, including the GWO algorithm, the SA algorithm, and the GWO-SA algorithm [32], thereby establishing the superiority of the greedy-genetic algorithm.

### Simulation experiment of container placement between virtual machines solved by greedy strategy

This experiment posits that a container task encompasses three distinct types of resource requests: CPU, Memory, and I/O bandwidth. The demand for each resource type is contingent upon the specific circumstances of the task. To enhance both the comprehensiveness and simplicity of the calculations, the requested amounts of CPU, Memory, and I/O are represented in units of *C* (units), *M* (GB), and *I* (Mbps), respectively. These amounts are aggregated into a weighted sum, referred to as *CT*_*SUM*, as delineated in Eq 10. This approach is designed to provide flexibility in resource allocation, allowing users to adjust the weights assigned to each resource type according to their particular requirements, thereby facilitating more rational and optimized resource distributions. Now specify that *α*_1_=0.4, *α*_2_=0.4, *α*_3_=0.2 in the formula.

$$\begin{array}{c} CT\_SUM=\alpha_{1}*C+\alpha_{2}*M+\alpha_{3}*I \end{array}$$ (10)

The running time time under the ideal state of a container task can be estimated by the performance data such as CPU, Memory and I/O required by the container task. Now give the CPU amount, Memory amount and I/O amount of 20 container task requests, which are required to be deployed on 4 virtual machines with the same configuration. The specific requirements are shown in Table 3.

**Table 3:** Resource requests and running times for each container.

	CPU/units	Memory/ GB	IO/Mbps	CT_SUM	Running time/s
Container 1	0.2	0.25	100	20.18	20.1
Container 2	0.6	1.02	400	80.65	20.7
Container 3	1.0	2.00	1000	201.20	16.5
Container 4	0.8	1.50	500	100.92	24.4
Container 5	0.1	0.10	50	10.08	16.0
Container 6	0.3	0.70	200	40.40	28.2
Container 7	0.5	1.20	300	60.68	32.3
Container 8	0.2	0.30	150	30.20	16.1
Container 9	1.2	2.50	1000	201.48	20.6
Container 10	0.7	1.00	350	70.68	23.2
Container 11	0.4	0.80	300	60.48	21.6
Container 12	0.1	0.20	80	16.12	20.0
Container 13	0.6	1.30	500	100.76	21.1
Container 14	0.3	0.50	100	20.32	40.2
Container 15	0.9	2.00	700	141.16	23.3
Container 16	0.9	1.2	500	100.84	19.7
Container 17	0.6	0.8	300	60.56	21.6
Container 18	0.8	1.5	600	120.92	20.4
Container 19	0.5	0.6	200	40.44	24.3
Container 20	1.0	1.8	1200	241.12	12.5

According to the greedy algorithm design, the specified numerical interval [*Res*_*min*_, *Res*_*max*_] of the virtual machine resource consumption obtained by Eqs 1 and 2 is [367.00,492.60], when the overall amount of resources utilized by each virtual machine falls within this range, it can be regarded as being in a fairly balanced state. At this time, according to the greedy algorithm scheme, the container number, total resource consumption deployed to 4 virtual machines are shown in Table 4.

**Table 4:** Consumed resources for each virtual machine.

	Container number	VM_SUM
VM 1	3,13,15	443.12
VM 2	7,10,11,20	432.96
VM 3	1,2,4,8,12,17,18	429.55
VM 4	5,6,9,14,16,19	413.56

**Fig 8:**
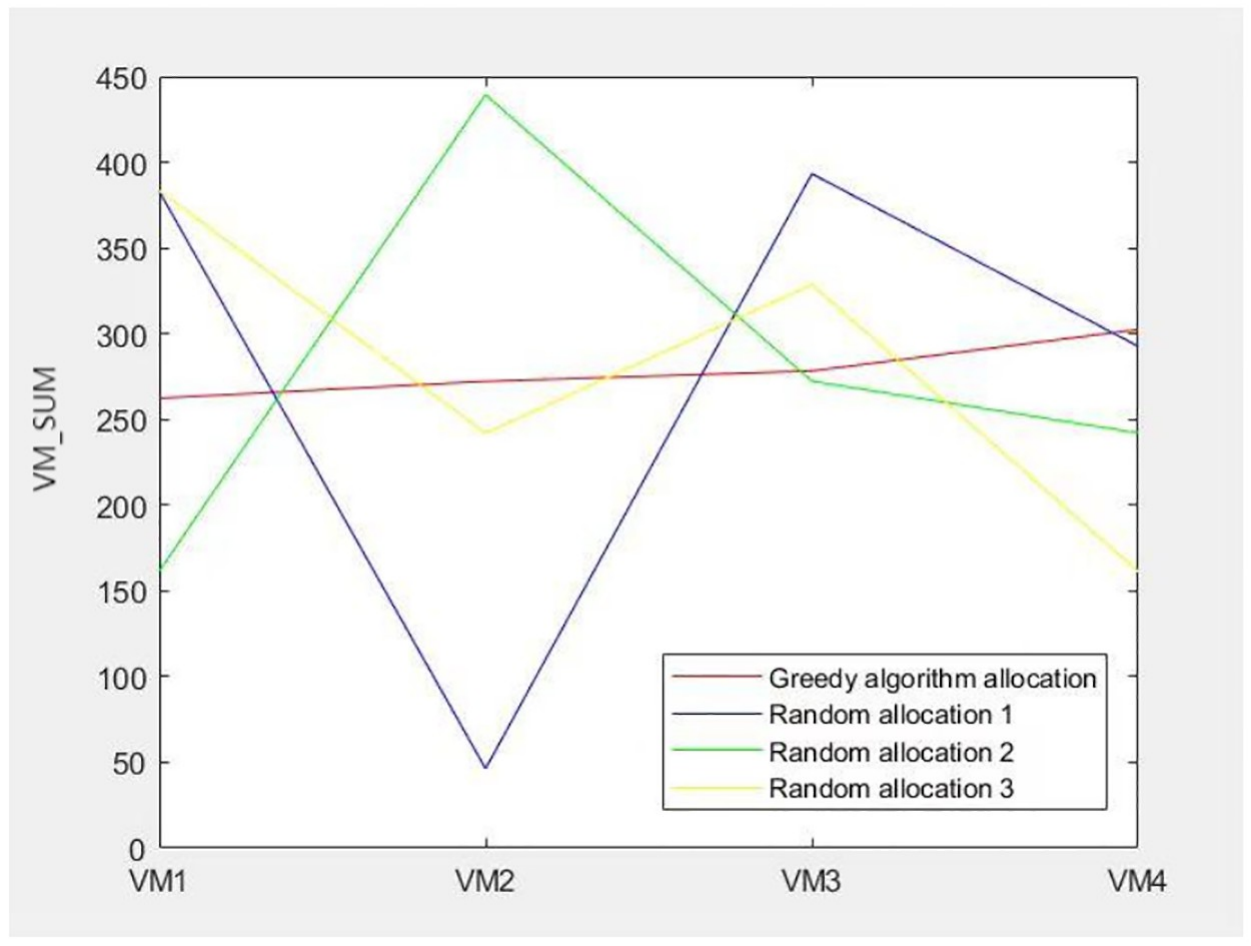
Total resource consumption of different allocation schemes.

The results of the algorithm design indicate that the *VM*_*SUM* values representing the total resource consumption for each virtual machine are relatively uniform, with none exhibiting excessive or insufficient resource usage. All values fall within the specified numerical range, thereby meeting the established requirements. In contrast, the total resource consumption for load balancing is significantly less effective when multiple container tasks are assigned and deployed randomly across the virtual machines, as compared to the outcomes produced by the greedy algorithm. The following Fig 8 clearly illustrates that the resource allocation scheme implemented by the greedy algorithm achieves a more equitable distribution of resources across each virtual machine, effectively preventing scenarios in which certain virtual machines are burdened with a disproportionate number of containers while others remain underutilized.

### Simulation experiment of resource allocation inside virtual machine solved by genetic algorithm

The above simulation experiment results indicate that four virtual machines are deployed, each with a different number of container tasks. When different container tasks are required to be deployed to a virtual machine, each container task has its own resource requirements. CPU, Memory and I/O requirements are different. The configuration of the four virtual machines is assumed to be identical, and the CPU, Memory and I/O resources that can be allocated are 2 units, 4GB and 2000Mbps respectively. The resource requirements of the four virtual machines are shown in Tables 5–8.

**Table 5:** The resource requirements of virtual machine 1.

	CPU/units	Memory/ GB	IO/Mbps	CT_SUM	Running time/s
Container 3	1.0	2.00	1000	201.20	16.5
Container 13	0.6	1.30	500	100.76	21.1
Container 15	0.9	2.00	700	141.16	23.3

**Table 6:** The resource requirements of virtual machine 2.

	CPU/units	Memory/ GB	IO/Mbps	CT_SUM	Running time/s
Container 7	0.5	1.20	300	60.68	32.3
Container 10	0.7	1.00	350	70.68	23.2
Container 11	0.4	0.80	300	60.48	21.6
Container 20	1.0	1.8	1200	241.12	12.5

**Table 7:** The resource requirements of virtual machine 3.

	CPU/units	Memory/ GB	IO/Mbps	CT_SUM	Running time/s
Container 1	0.2	0.25	100	20.18	20.1
Container 2	0.6	1.02	400	80.65	20.7
Container 4	0.8	1.50	500	100.92	24.4
Container 8	0.2	0.30	150	30.20	16.1
Container 12	0.1	0.20	80	16.12	20.0
Container 17	0.6	0.8	300	60.56	21.6
Container 18	0.8	1.5	600	120.92	20.4

**Table 8:** The resource requirements of virtual machine 4.

	CPU/units	Memory/ GB	IO/Mbps	CT_SUM	Running time/s
Container 5	0.1	0.10	50	10.08	16.0
Container 6	0.3	0.70	200	40.40	28.2
Container 9	1.2	2.50	1000	201.48	20.6
Container 14	0.3	0.50	100	20.32	40.2
Container 16	0.9	1.2	500	100.84	19.7
Container 19	0.5	0.6	200	40.44	24.3

**Fig 9:**
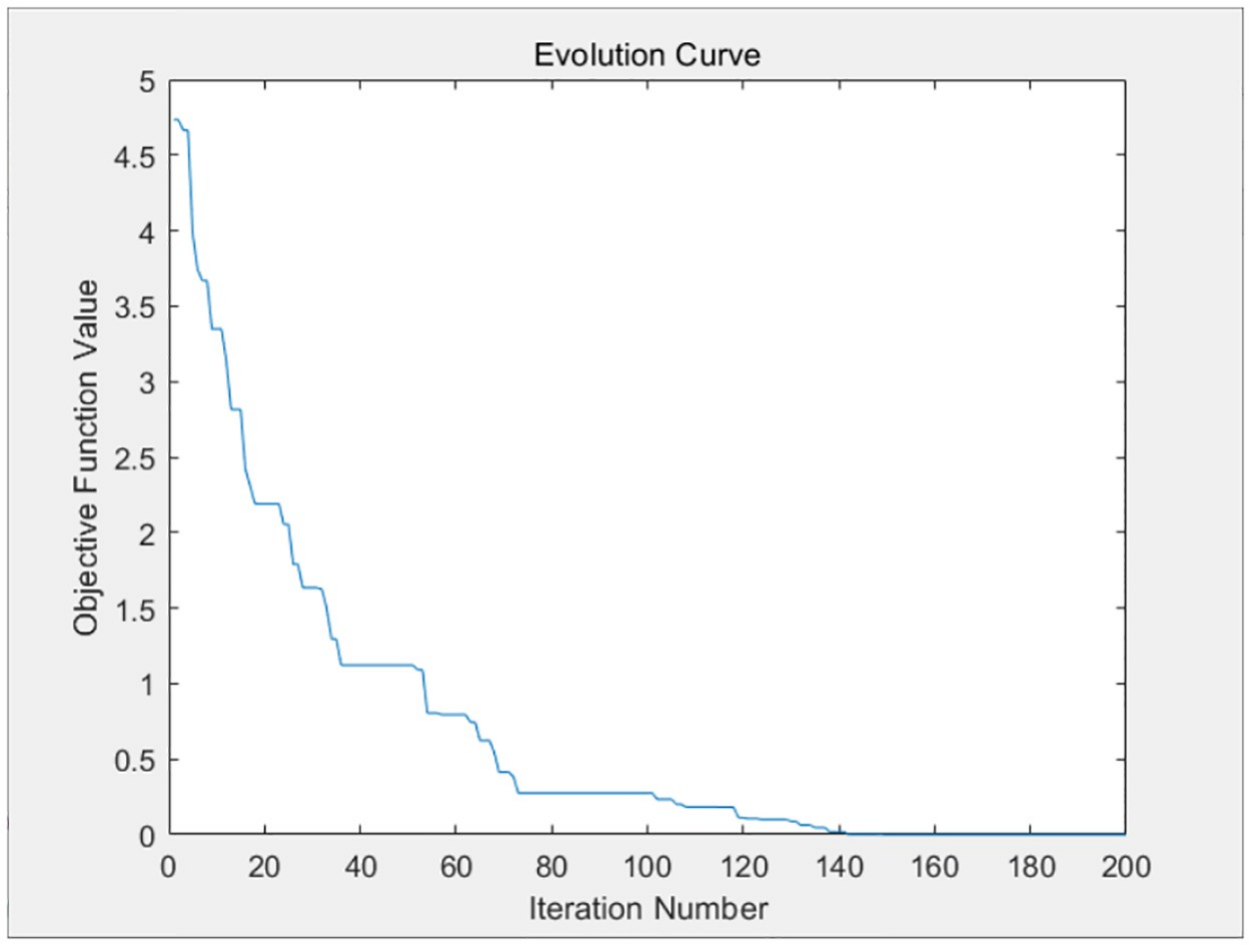
The iteration curve of virtual machine 1.

**Fig 10:**
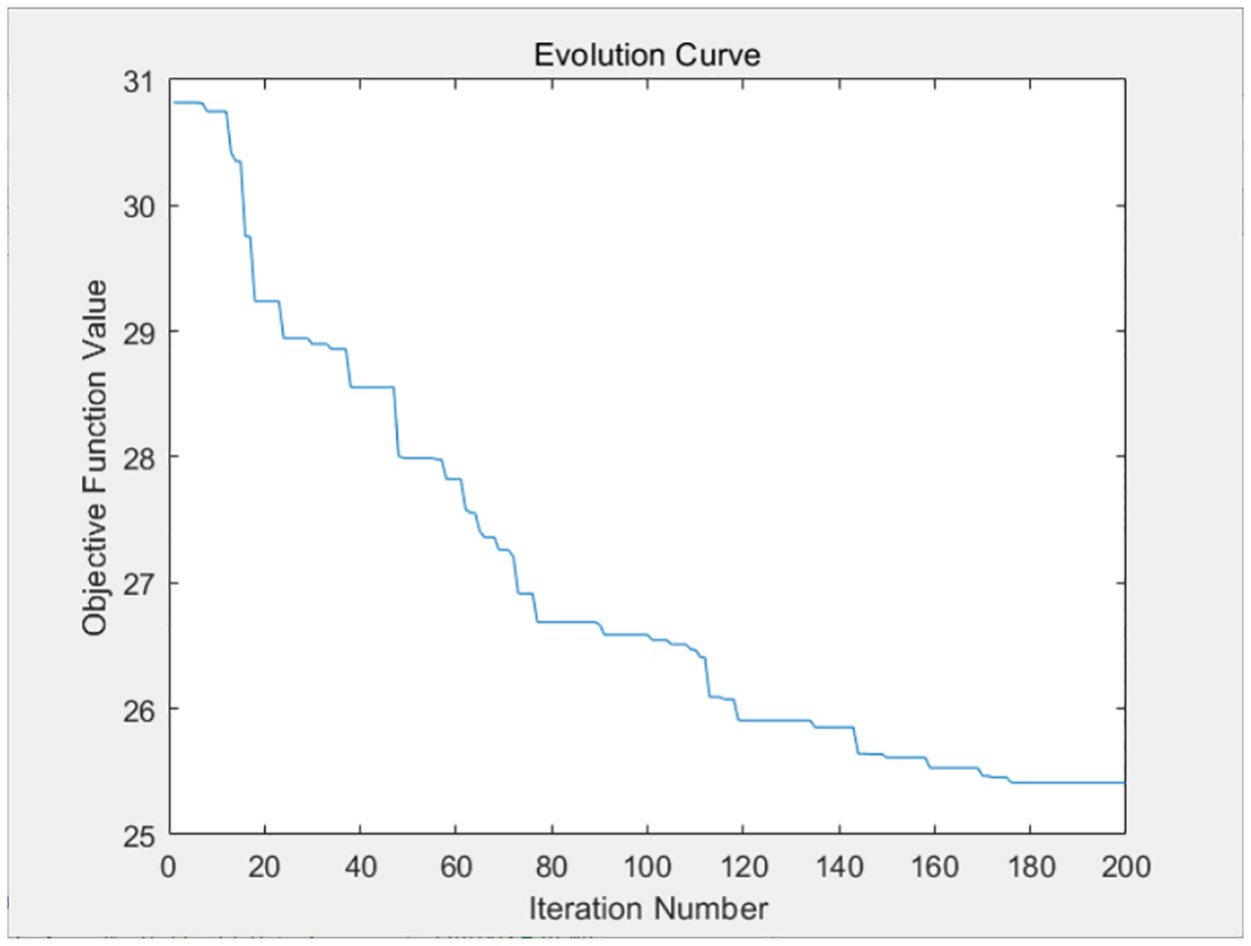
The iteration curve of virtual machine 2.

**Fig 11:**
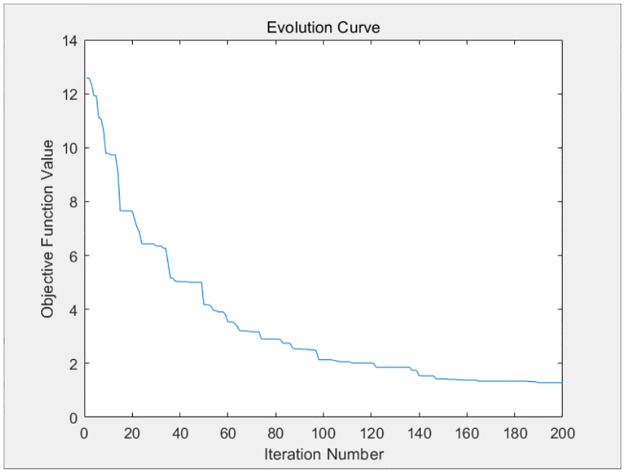
The iteration curve of virtual machine 3.

**Fig 12:**
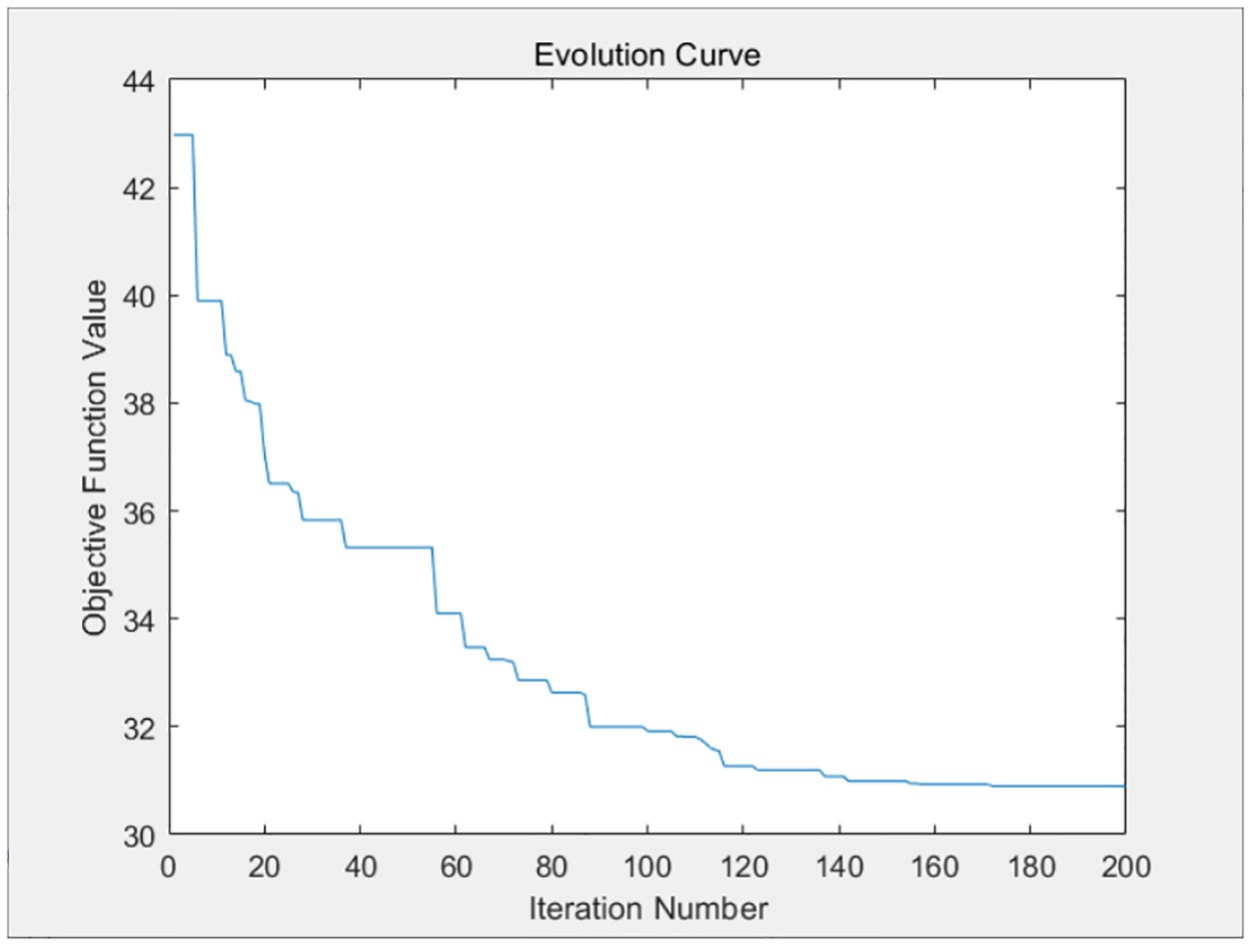
The iteration curve of virtual machine 4.

The three resource requests of each container task of the above four virtual machines are taken as parameters and substituted into the genetic algorithm design of this paper. The genetic algorithm will distribute the resources optimally to each container based on the available resources of the virtual machine, so as to balance the completion time of each container task, and avoid large discrepancies in the completion time of each container on each virtual machine. The fitness evolution curve obtained by the genetic algorithm after 200 iterations is shown in Figs 9–12.

As shown in Figs 9–12, the variance of the container time on each virtual machine decreases gradually as the number of iterations increases, and the completion time of each container tends to be balanced. The completion time of each container on each virtual machine is given as follows.

**Fig 13:**
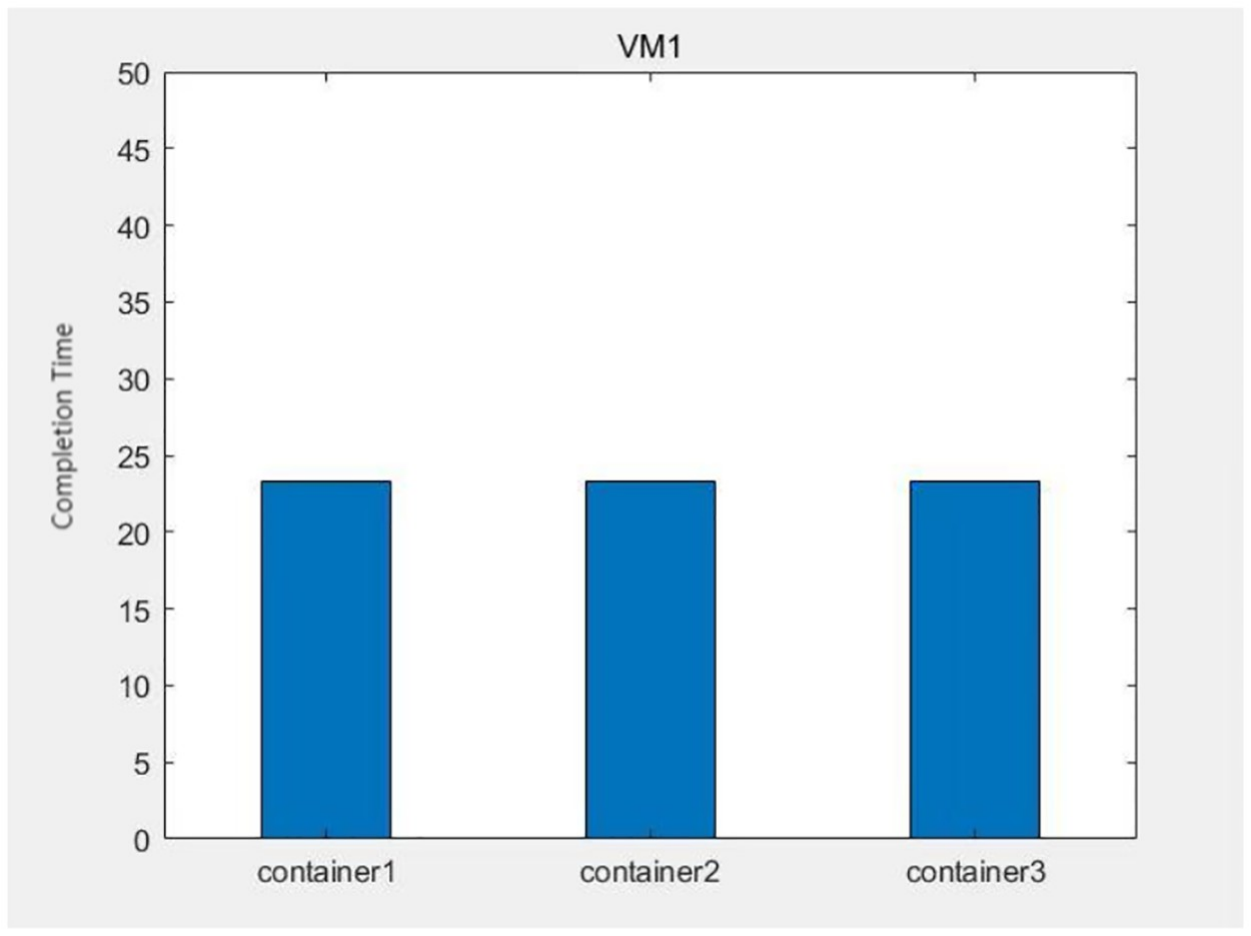
The container task completion time on virtual machine 1.

**Fig 14:**
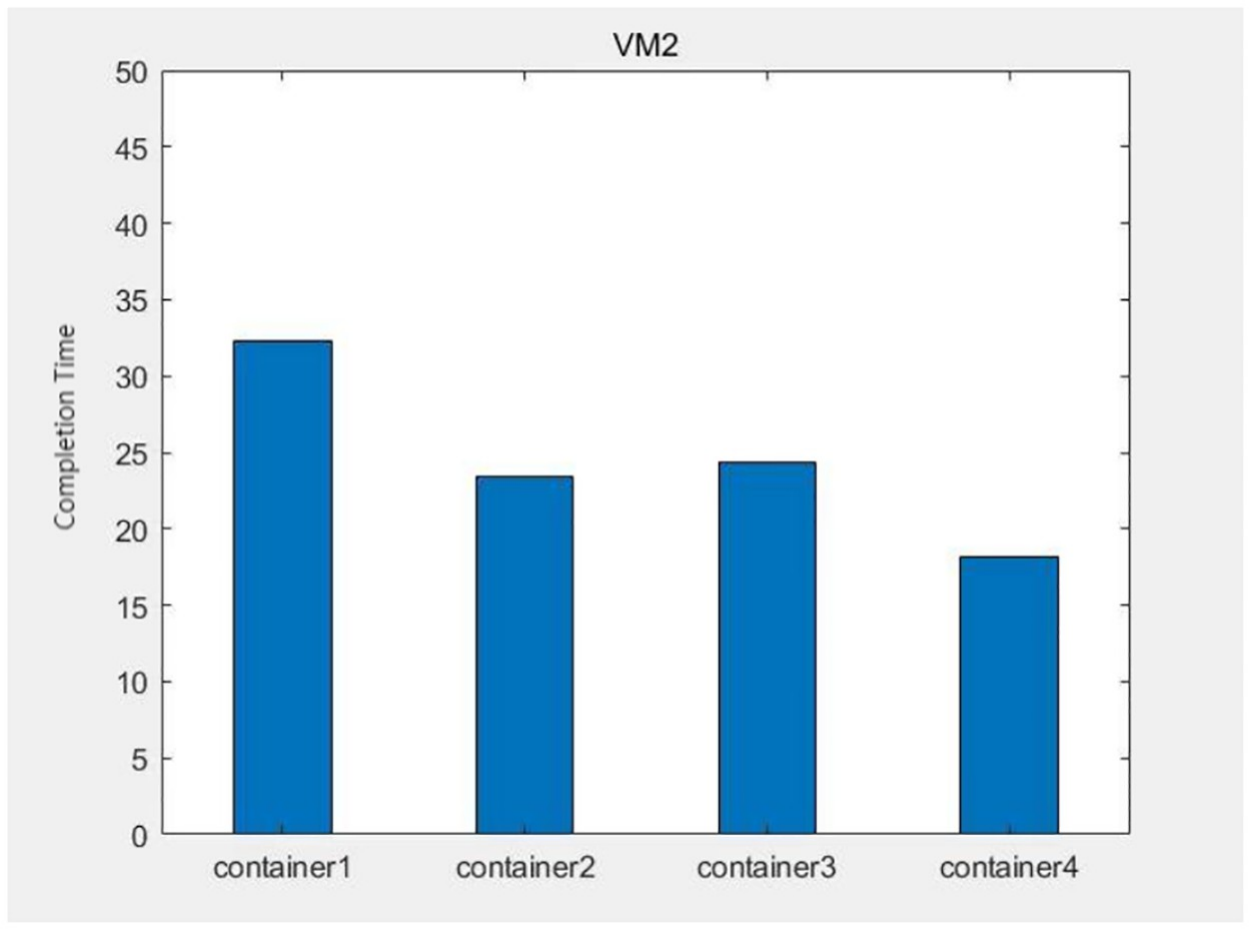
The container task completion time on virtual machine 2.

**Fig 15:**
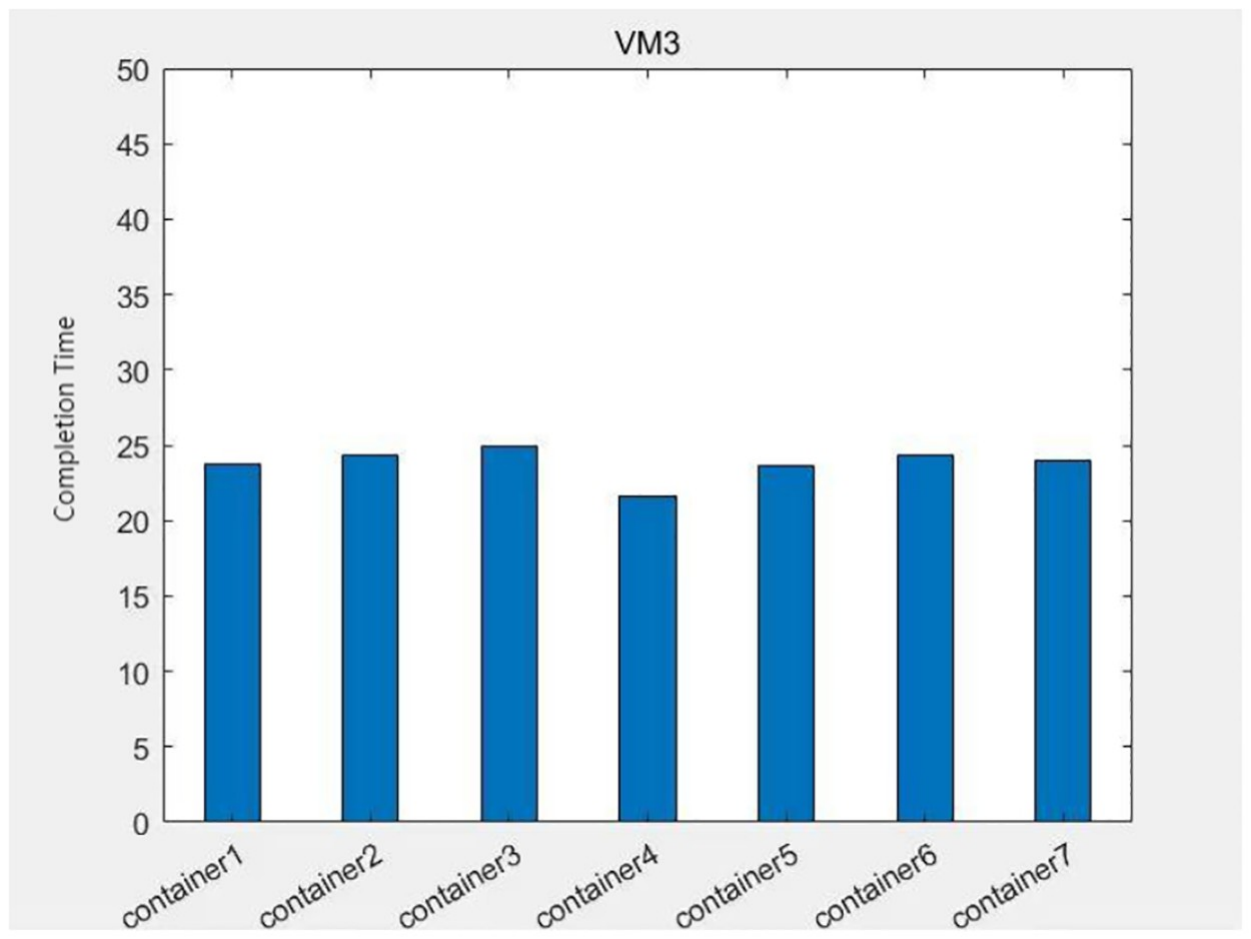
The container task completion time on virtual machine 3.

**Fig 16:**
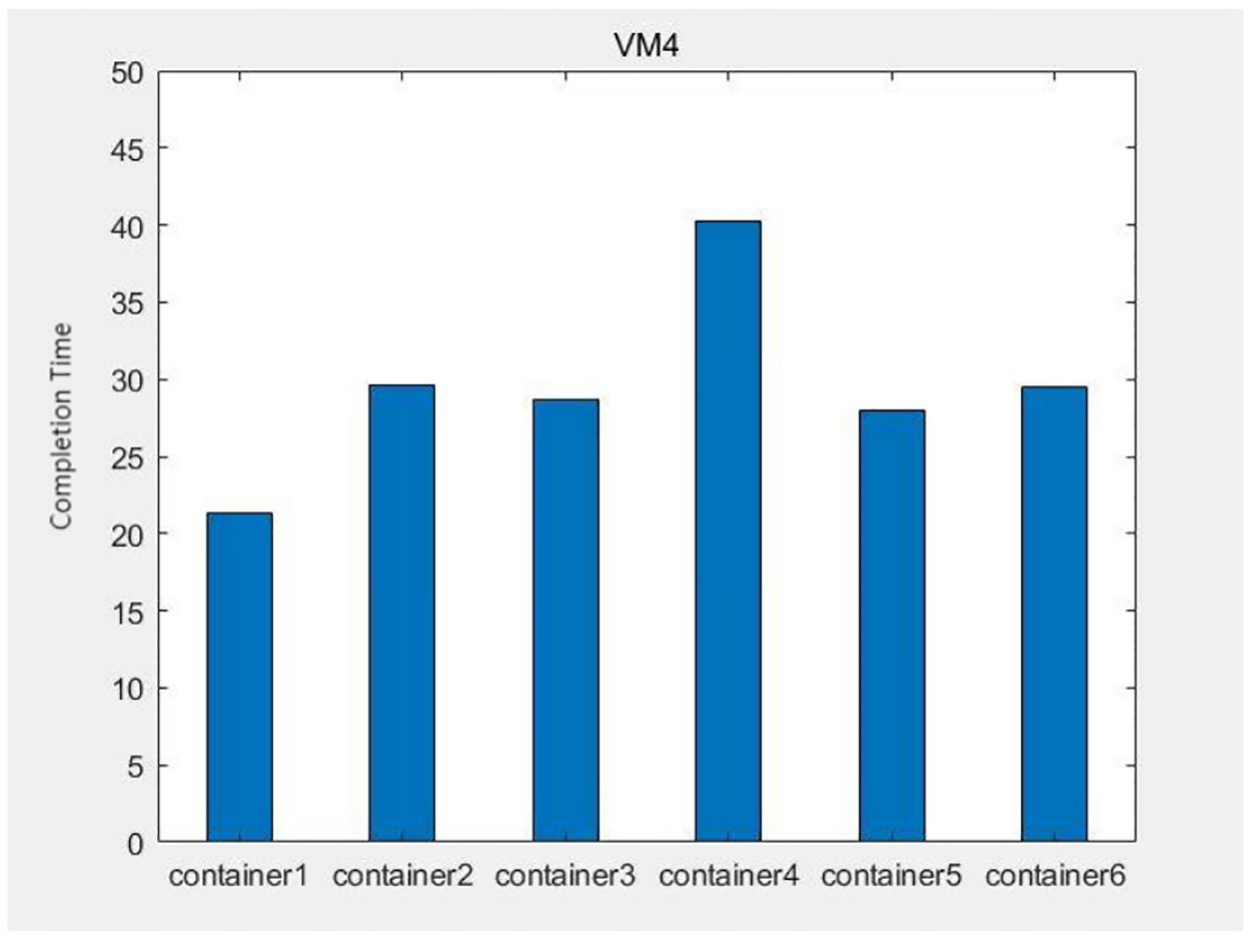
The container task completion time on virtual machine 4.

The data from Figs 13–16 shows that after each virtual machine allocates three kinds of resources to each container reasonably by the genetic algorithm, the completion time of each container is relatively balanced, except for some containers such as container 4 on virtual machine 4, which takes too long to complete due to its large computational task compared to other containers. The time difference degree of other containers is small, which means that the completion time dispersion is low after the virtual machine balances the resources for each container, that is, the completion time does not differ much, thus making the subprocess task finish in the shortest time.

### Comparison of container deployment algorithms between two-stage hybrid allocation and algorithms for other heuristics allocation

The aforementioned algorithms illustrate that the deployment scheme utilizing a greedy algorithm is capable of appropriately allocating multiple container tasks with diverse resource request sizes to each virtual machine. This approach results in a more equitable distribution of resource consumption across the virtual machines. The genetic algorithm allocation scheme facilitates the distribution of resources to each container based on the inherent resources of the virtual machine, thereby balancing the completion times of each container and ultimately achieving the minimum completion time for the subprocess task on the virtual machine.

The advantage of the two-stage hybrid algorithm presented in this paper lies in its ability to effectively address the challenge of deploying multiple container tasks across various virtual machines. Initially, the algorithm balances the allocation of multiple containers to each virtual machine based on their resource consumption. Subsequently, it ensures the provision of sufficient resources for the execution of these containers within the virtual machine that accommodates multiple container tasks. This approach ultimately enhances the efficiency of completing the entire subprocess task.

The simulated annealing (SA) optimization algorithm exhibits significant generality and robustness in addressing resource allocation problems, effectively managing complex nonlinear optimization challenges. Nevertheless, it is characterized by a relatively slow convergence rate and a sensitivity to initial conditions and parameter settings, which can impede the rapid identification of the global optimal solution.

The Grey Wolf Optimization (GWO) algorithm is widely regarded as an effective approach for addressing resource allocation problems, owing to its straightforward structure and rapid convergence capabilities. These characteristics facilitate a balance between local search and global optimization. Nonetheless, the GWO algorithm is not without its limitations; it is susceptible to premature convergence, which can result in the algorithm settling on a local optimal solution during the search process, thereby potentially neglecting the identification of the global optimal solution.

The simulated annealing algorithm, which is based on grey wolf optimization (GWO-SA), seeks to integrate the strengths of both methodologies to enhance the efficiency and accuracy of resource allocation problem-solving. This algorithm has the potential to mitigate premature convergence through the simulated annealing process, while simultaneously capitalizing on the rapid convergence characteristics of the grey wolf optimization algorithm to expedite the search process. Nevertheless, the amalgamation of these two algorithms may lead to increased implementation complexity and computational costs, necessitating more meticulous parameter tuning to achieve an optimal balance in performance.

**Fig 17:**
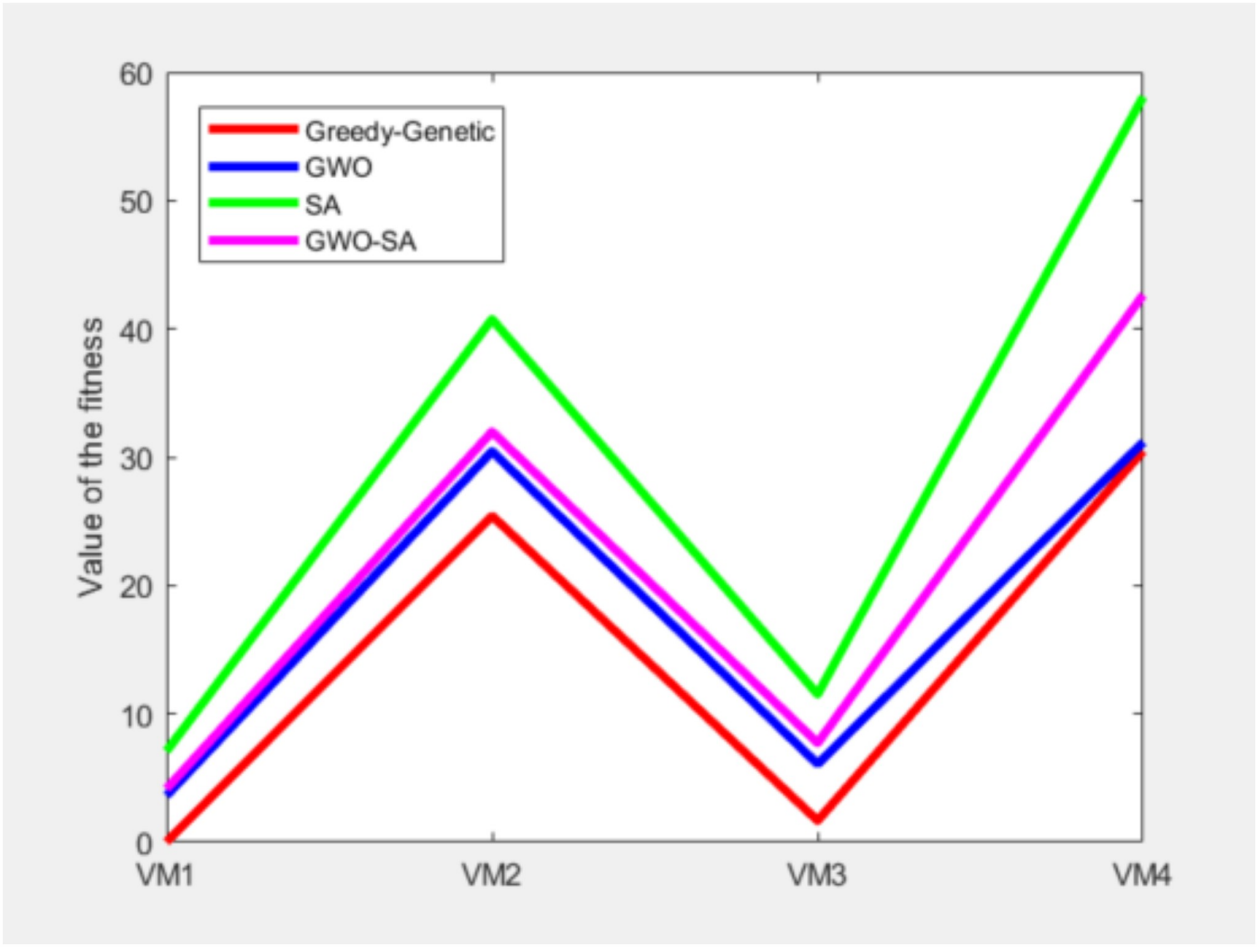
The fitness value for each algorithm to execute under four virtual machines.

Now, based on the problem proposed in “Simulation experiment of container placement between virtual machines solved by greedy strategy” that containers 1-20 are required to be deployed to virtual machines 1-4, a comparison of the Greedy-Genetic algorithm and the GWO algorithm and the SA algorithm and the GWO-SA algorithm, as shown in Fig 17.

**Fig 18:**
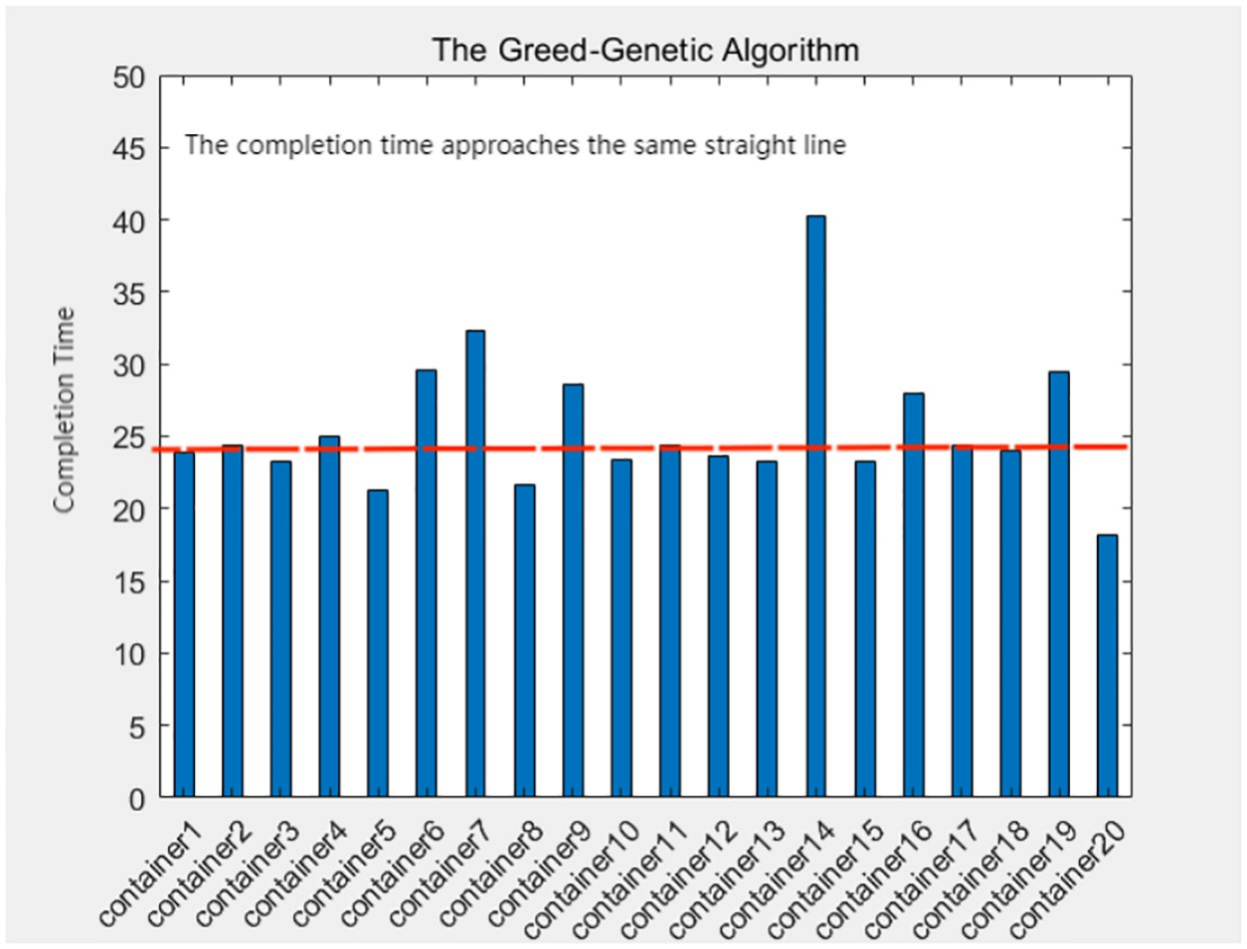
The completion time of each container in the Greed-Genetic algorithm.

**Fig 19:**
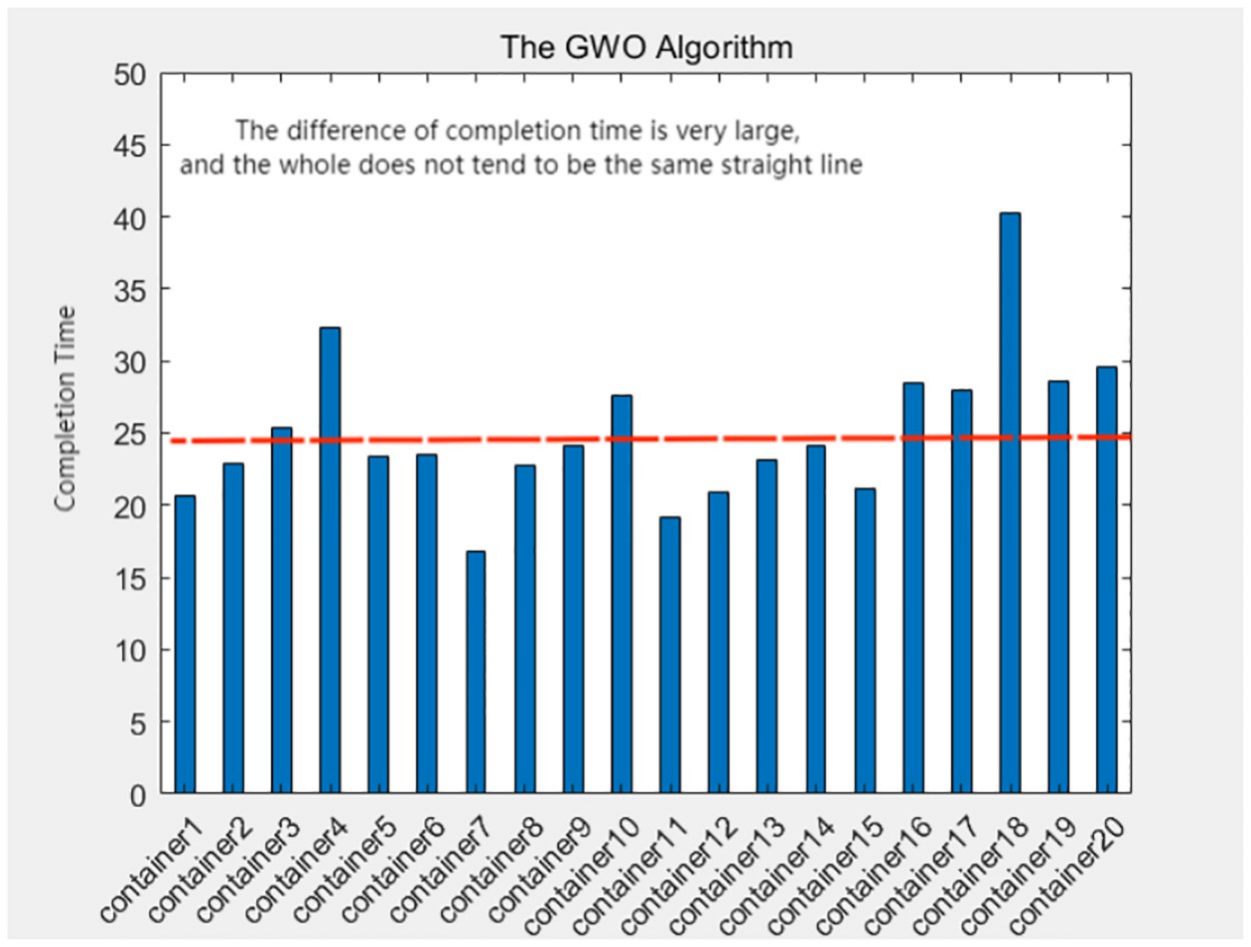
The completion time of each container in the GWO algorithm.

**Fig 20:**
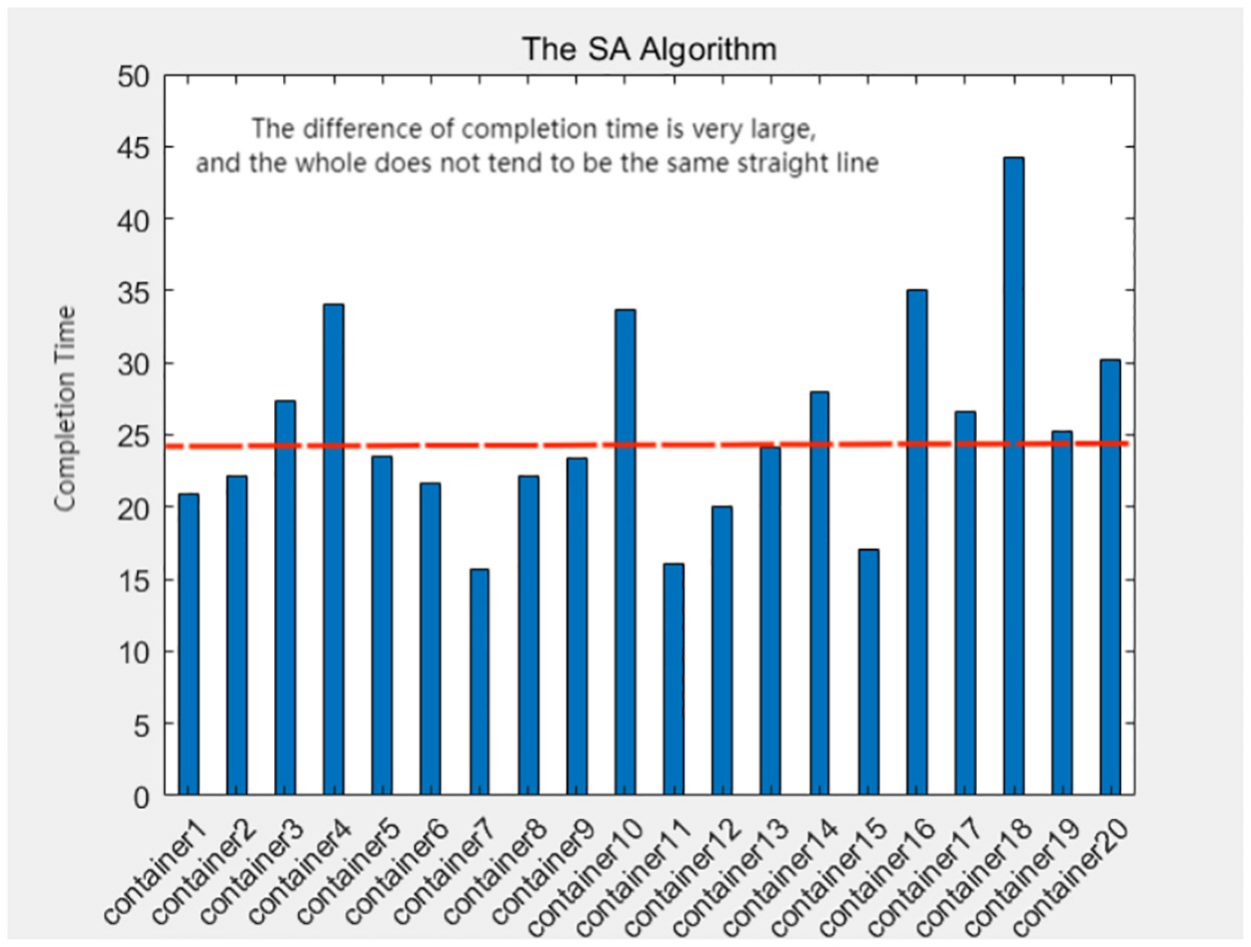
The completion time of each container in the SA algorithm.

**Fig 21:**
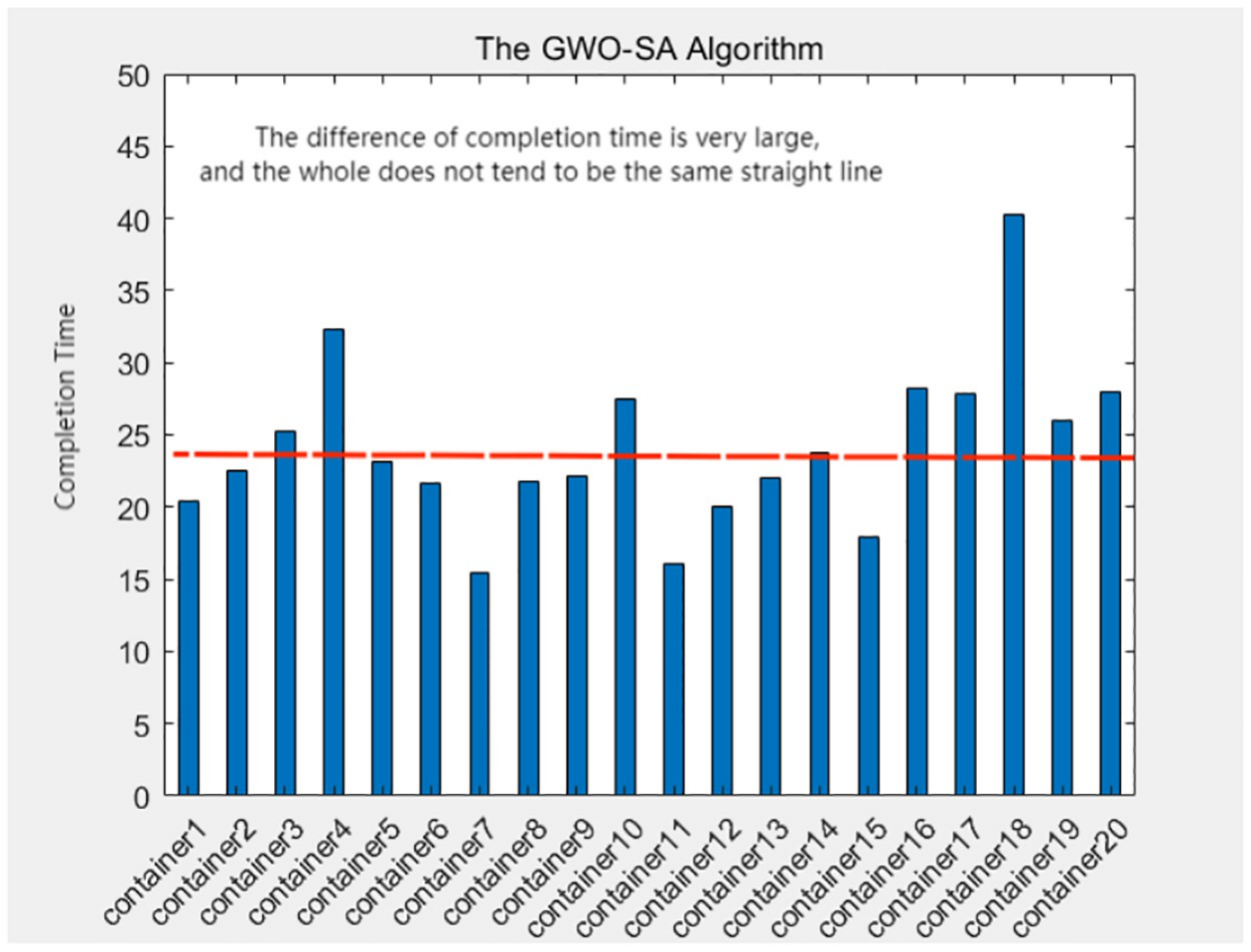
The completion time of each container in the GWO-SA algorithm.

A comprehensive analysis of the completion times for all container tasks indicates that the allocation scheme utilized by the greedy-genetic algorithm demonstrates a notable degree of balance in the completion times of the majority of containers. In contrast, the other three heuristic algorithms exhibit considerable disparities in completion times among individual containers. This finding underscores the effectiveness of the greedy-genetic approach in achieving a nearly uniform distribution of workload across containers, thereby improving overall resource utilization and system efficiency. The balanced completion times not only facilitate a smoother workflow but also mitigate the risk of resource bottlenecks and delays, rendering it a preferable solution for resource allocation in complex containerized environments. As shown in Figs 18–21.

**Fig 22:**
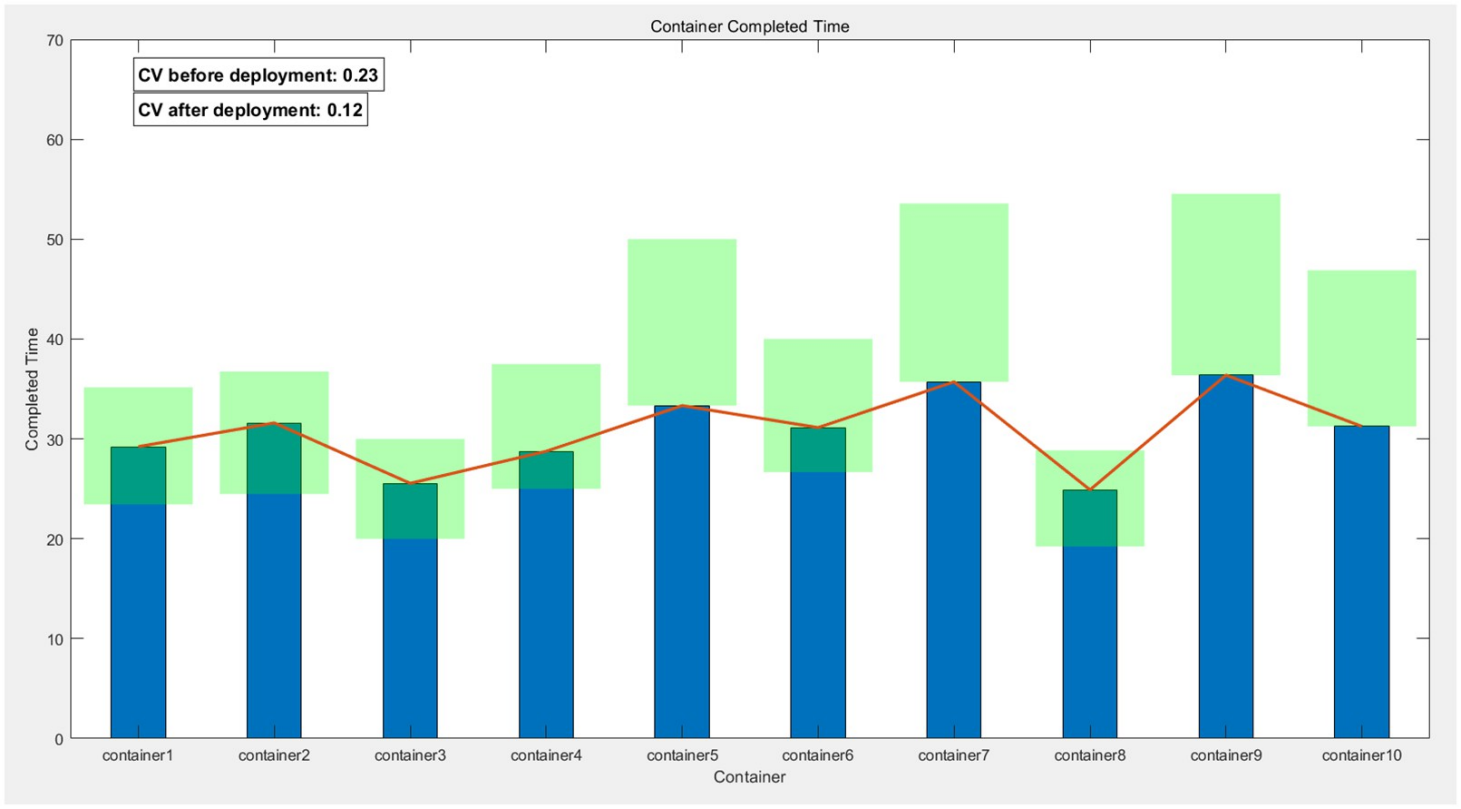
Balanced completion times of 10 containers and comparison.

**Fig 23:**
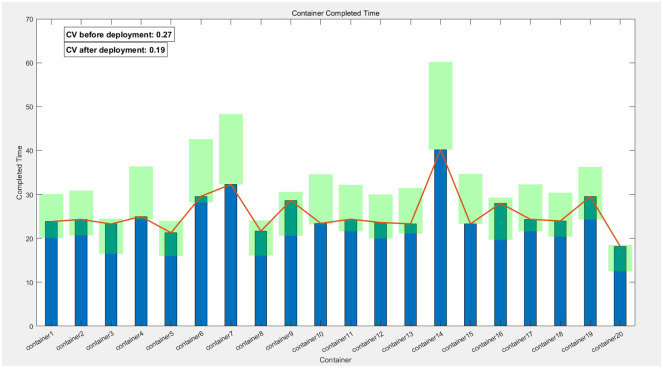
Balanced completion times of 20 containers and comparison.

**Fig 24:**
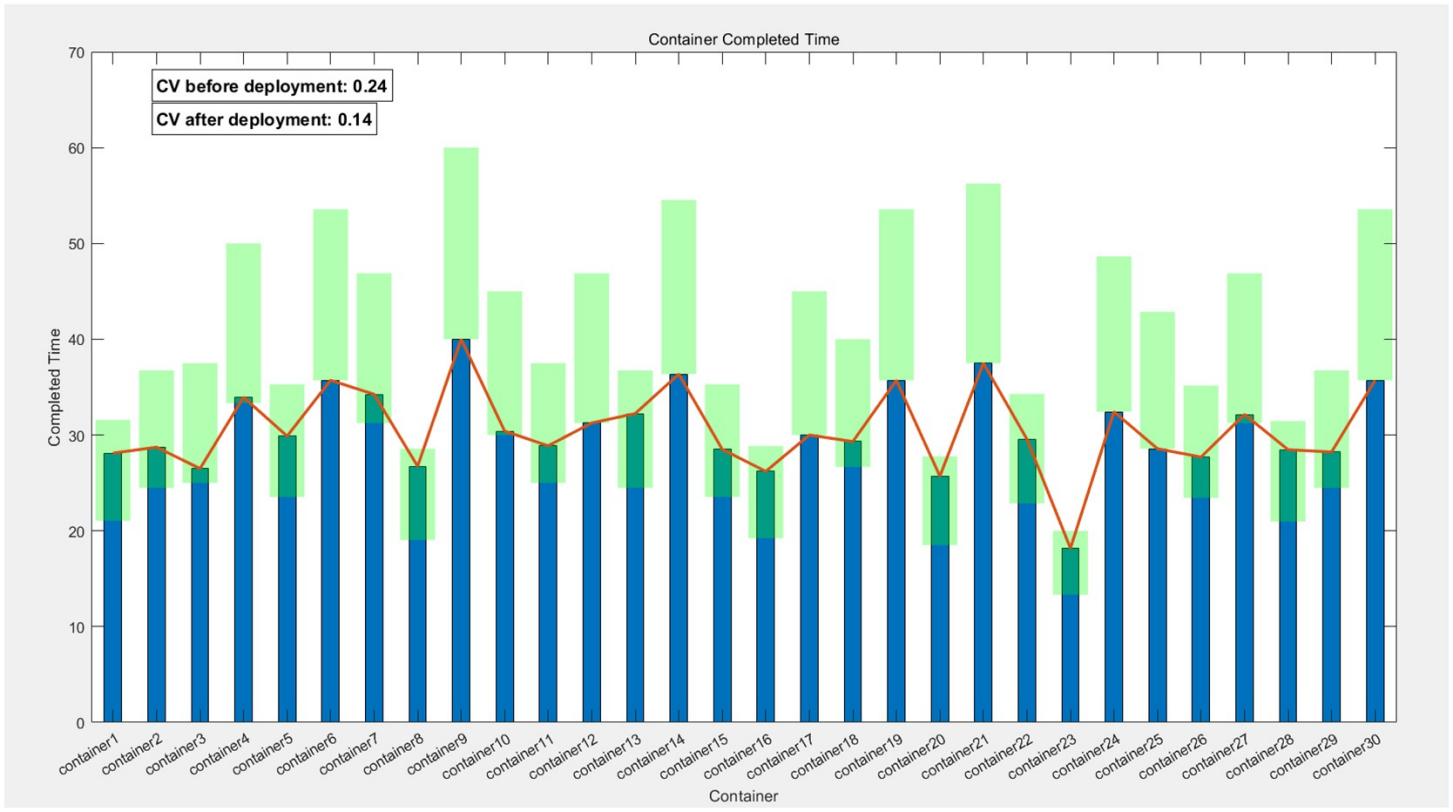
Balanced completion times of 30 containers and comparison.

In this chapter, to ensure the rationality and reproducibility of the experimental design, we have not only meticulously detailed the process and outcomes of applying the Greedy-Genetic algorithm for resource allocation and deployment balancing among 20 containers but also expanded the experimental scope by conducting corresponding validations on 10 and 30 containers, respectively. The experimental results are presented in Figs 22–24. In these figures, the green-filled areas represent the lower and upper bounds of the runtime intervals for each container. Through observation, it is evident that the resource allocation strategy optimized by the Greedy-Genetic algorithm results in a uniform distribution of completion times for all containers within a relatively narrow range, thereby minimizing the overall task completion time.

Furthermore, we conducted a comparative analysis of the fastest completion times for each container before deployment and the coefficient of variation (CV, the ratio of the standard deviation to the mean, used to quantify the dispersion of data distributions) of the completion times after deployment. The CV serves as an indicator of data distribution concentration, with a lower CV indicating a more centralized and balanced distribution. Our comparative analysis revealed a significant reduction in the CV of completion times for all containers after deployment compared to before. This finding suggests that, through optimized deployment, the completion times of the containers exhibit a more balanced distribution, indicating a significant improvement in the balance of completion times. Not only does this validate the effectiveness of the Greedy-Genetic algorithm in addressing resource allocation problems, but it also provides robust data support and theoretical justification for subsequent related research.

## Summary and future work

This paper presents a two-stage optimization method for load balancing in container deployment resource allocation, aimed at addressing the issues of resource allocation imbalance and inefficiency prevalent in existing methodologies. The first stage of the proposed method involves the systematic allocation of container requests, which require specific resources, to multiple virtual machines. This allocation is conducted in a manner that is both rational and efficient. By modeling the resource demands of each container, the method calculates the intervals of relative balance in resource consumption for each virtual machine. Subsequently, a greedy algorithm is employed to partition these intervals into optimal allocation combinations, facilitating the deployment of multiple containers accordingly. Upon completion of the preliminary placement in the first stage, the second stage focuses on balancing the inherent resources of individual virtual machines among the container requests. This is achieved through a modeling calculation of the utilization rates of allocated resources for each container, alongside the corresponding budgeted time. The method conceptualizes the allocated resources as genes and the allocation schemes as chromosomes. It then utilizes genetic algorithm operations, including initialization, evolution, crossover, and mutation, to derive the optimal resource allocation scheme. Furthermore, this paper conducts a comparative evaluation of deploying 20 containers across 4 virtual machines, utilizing both the proposed two-stage load balancing optimization method and three alternative heuristic algorithms. The results, which reflect the relative balance of completion times for each algorithm, indicate that the optimization deployment method introduced in this study demonstrates superior efficiency and balance.

In the future, we will conduct and expand our research from the following perspectives:

(1)Algorithm Optimization: This paper proposes to enhance the modeling and calculation of the resource utilization rate of containers by incorporating a more realistic resource matching similarity rate for future analysis and computation.
(2)Remaining Resource Allocation: Following the implementation of various load balancing resource allocation schemes, the genetic algorithm proposed in this study will inevitably result in some unallocated resources. In future research, we will explore the application of a more systematic and equitable approach to distribute these remaining resources among each container, thereby enhancing the overall resource utilization rate.
(3)Real Case Study: In future research, we will explore the implementation of a real-world small-scale application-level container deployment problem. This will allow us to compare its effectiveness against traditional deployment schemes and to conduct a thorough evaluation and verification of the results.
